# Development and content validity assessment of the Dry Eye Disease Questionnaire in patients with dry eye disease, meibomian gland dysfunction, and Sjögren’s syndrome dry eye disease

**DOI:** 10.1186/s41687-023-00608-5

**Published:** 2023-07-05

**Authors:** Brigitte Sloesen, Alyson Young, Katie Forde, Nicola Hodson, Sarah Bentley, Oonagh Walsh, Christel Naujoks, Paul O’Brien, Garima Sharma

**Affiliations:** 1 Novartis Pharma AG, Basel, Switzerland; 2 Adelphi Values, Patient-Centered Outcomes, Cheshire, UK; 3 Novartis Ireland Limited, Dublin, Ireland; 4 Novartis Healthcare Private Limited, Hyderabad, India

**Keywords:** Dry eye disease, Meibomian gland dysfunction, Sjogren’s syndrome dry eye, Patient-reported outcome, Concept elicitation, Cognitive debriefing, Content validity

## Abstract

**Background:**

Dry eye disease (DED), Meibomian gland dysfunction (MGD), and Sjögren’s syndrome dry eye disease (SS-DED) are eye dryness conditions that show significant overlap in various symptoms of ocular discomfort. The aim of this study was to qualitatively explore the patient experience and evaluate content validity of the newly developed Dry Eye Disease Questionnaire (DED-Q).

**Methods:**

Semi-structured interviews were conducted with 61 US adults who reported experiencing ocular symptoms due to their physician-confirmed primary diagnosis of DED (n = 21), MGD (n = 20), or SS-DED (n = 20). The open-ended concept-elicitation phase was followed by cognitive debriefing (CD) of the DED-Q to evaluate participants’ understanding and relevance of the instructions, items, response options, and recall periods. Interviews were also conducted with eight specialist healthcare professionals to assess clinical relevance of the concepts included. Verbatim interview transcripts were analyzed using thematic analysis in ATLAS.ti v8 software.

**Results:**

A total of 29 symptoms and 14 impacts on quality of life were reported across participant interviews. Primary ocular symptoms reported included eye dryness (n = 61/61; 100%), eye irritation (n = 55/61; 90%), eye itch (n = 54/61; 89%), burning sensation (n = 52/61; 85%), and foreign body sensation (n = 51/61; 84%). The most impacted aspects of daily life were using digital screens (n = 46/61; 75%), driving (n = 45/61; 74%), working (n = 39/61; 64%), and reading (n = 37/61; 61%). CD findings showed most participants had good understanding of DED-Q items and confirmed most concepts were relevant to the lived experience of their condition. Aside from few minor changes to the items and examples to facilitate more accurate interpretation, the proposed instruction wording was modified for various symptom and impact modules to encourage participants to focus only on dry eye vision problems.

**Conclusions:**

This research identified multiple prevalent symptoms and impacts of DED, MGD, and SS-DED, most of which were similar across the conditions. The DED-Q was confirmed to be a content-valid PRO measure suitable for use in clinical studies to assess the patient experience of DED, MGD, and SS-DED. Future work will focus on evaluating the psychometric properties of the DED-Q for use as an efficacy endpoint in clinical trials.

**Supplementary Information:**

The online version contains supplementary material available at 10.1186/s41687-023-00608-5.

## Abstract (plain language summary)

Dry Eye Disease (DED), Meibomian Gland Dysfunction (MGD), and Sjögren’s Syndrome Dry Eye Disease (SS-DED) are conditions related to dryness of the eye. There is no suitable patient-reported outcome (PRO) measure available for use across the three conditions. Interviews were conducted with 61 participants with any of the three conditions to understand the symptoms they experience and their impact on daily life. The findings were used to refine the content of a new PRO measure, the Dry Eye Disease Questionnaire (DED-Q). To evaluate the suitability of the DED-Q, participants were also asked to complete each item of the DED-Q during the interview and share their understanding of the item wording, response options and the recall period. Interviews were also conducted with eight healthcare professionals (HCPs). The main symptoms reported by the patients across three conditions were eye dryness, eye irritation, eye itch, burning sensation, and foreign body sensation. The symptoms caused difficulties in performing activities such as using digital screens, driving, working, and reading. Almost all participants demonstrated good understanding of the items on the DED-Q and found them to be relevant to their experience of their condition. The HCPs also confirmed that the concepts covered in the DED-Q were relevant to assess the participant experience across these conditions. Next steps will involve a more detailed analysis of the measurement properties of the DED-Q to confirm its usefulness in supporting primary or secondary endpoints in clinical trials.

**Supplementary Information:**

The online version contains supplementary material available at 10.1186/s41687-023-00608-5.

## Background

Dry eye disease (DED), Meibomian gland dysfunction (MGD), and Sjögren’s syndrome dry eye disease (SS-DED) are conditions associated with symptoms of ocular discomfort caused by inadequate lubrication of the eye. DED is a disorder of the tear film due to either deficiency of lubrication (aqueous-deficient DED) or excessive evaporation (evaporative DED) [1–4]. Evaporative DED can be caused by MGD [5–8] which is a chronic obstruction or functional abnormality of the meibomian glands responsible for secreting meibum, an oily substance that prevents evaporation of the eye’s tear film. Aqueous-deficient DED can be caused by SS-DED [9–11], which is a chronic autoimmune disease primarily affecting the salivary and tear glands, resulting in extensive dryness. Furthermore, evaporative and aqueous-deficient DED are not mutually exclusive and often co-exist contributing to the complexity of the disease.

According to the Tear Film & Ocular Surface Society (TFOS) Dry Eye Workshop II (TFOS DEWS II) epidemiology report, the prevalence of DED is estimated to be between 5 and 50% of the general population and ultimately unknown, with the prevalence of mild or episodic DED tending to be higher than that of severe forms of the disease [12]. The prevalence of MGD varies in the literature; however, research has found that MGD is typically higher in Asian populations (range: 46%-70%) than in Caucasian populations (range: 3.5%-20%) [13–15]. Older individuals are at a greater risk of developing MGD. The prevalence of SS-DED is estimated to be approximately 0.6% (range: 0.19–1.39%). In the United States (US), the incidence of physician-diagnosed SS-DED among the White population has been reported to be 3.9 per 100,000 patients per year, with a 14 × higher rate among women than among men [2].

The clinical manifestations of DED, MGD, and SS-DED can be highly variable. Key symptoms of these conditions overlap significantly and include eye dryness, grittiness, burning, pain, irritation, redness, inflammation, itchiness, and scratchy and stingy eyes [16, 17]. Overall, while there is limited published evidence relating to the patient experience of DED, MGD, and SS-DED, studies show that these symptoms can cause patients to experience difficulties with daily tasks that rely on vision, such as using digital screens, reading, and driving, which can significantly impact the physical, emotional, and social domains of patients’ Health-Related Quality of Life (HRQoL) [12, 18–20].

A patient-reported outcome (PRO) is a subjective report that comes directly from the patient without the input or interpretation of a clinician or any other healthcare professional. PRO measures provide unique insight into the patient experience of the disease symptoms and impacts that cannot be adequately measured through routine objective assessments, and for this reason, are increasingly being incorporated into clinical and real-world evidence studies [21, 22]. The US Food and Drug Administration (FDA) PRO Guidance to Industry (2009) provides detailed recommendations on the scientific evidence required to achieve PRO labeling claims, clearly stipulating the need for patient input and feedback in the item generation and refinement process [23–26].

In line with the FDA PRO guidance, a targeted literature search was first conducted to identify existing PROs appropriate for use in DED, MGD, and SS-DED. None of the instruments identified were adequately fit-for-purpose [23]. Specifically, some of the instruments lacked evidence of content validity (i.e., developed without direct input from patients or not formally tested with patients via cognitive debriefing interviews), e.g., the Symptom Assessment in Dry Eye (SANDE), Standard Patient Evaluation of Eye Dryness questionnaire (SPEED), Dry Eye Questionnaire (DEQ), Ocular Comfort Index (OCI) [27–30]. Conceptual coverage in terms of the symptoms and ability to perform visual tasks was also limited for some of the measures, e.g., the Ocular Surface Disease Instrument (OSDI) does not assess severity/intensity of symptoms and is missing key dry eye symptoms, including foreign body sensation and tearing [31]. Another valuable and widely used PRO measure, the Impact of Dry Eye on Everyday Life (IDEEL) does not include items related to night-time driving and those related to intense digital technology and screen use relevant to the modern times [32]. Several PROs also tended to employ recall periods unsuitable for clinical trials (‘e.g., ‘last week’, ‘last two weeks’), which could introduce recall bias, thus preventing a comprehensive capture of the participant experience of their dry eye condition. Given the lack of instruments with robust evidence of the critical psychometric properties (i.e., evidence of reliability, validity, and responsiveness) for use in DED, MGD, and SS-DED, additional qualitative research was undertaken to further understand participant experience across the three conditions.

The aim of this study was to explore the lived experience of DED, MGD, and SS-DED participants in terms of the most frequent and bothersome symptoms and their impact on daily living to inform the potential adaptation of a newly developed PRO measure—the Dry Eye Disease Questionnaire (DED-Q)—and to generate evidence for its content validity to support clinical trial endpoints evaluating patient-perceived benefit from new therapies in DED, MGD, and SS-DED. In addition to the DED-Q, Patient Global Impression of change (PGI-C) and severity (PGI-S) items were developed for use in DED, MGD, and SS-DED participants and their appropriateness was evaluated during the interviews.

## Methods

### Study design

**Fig. 1:**
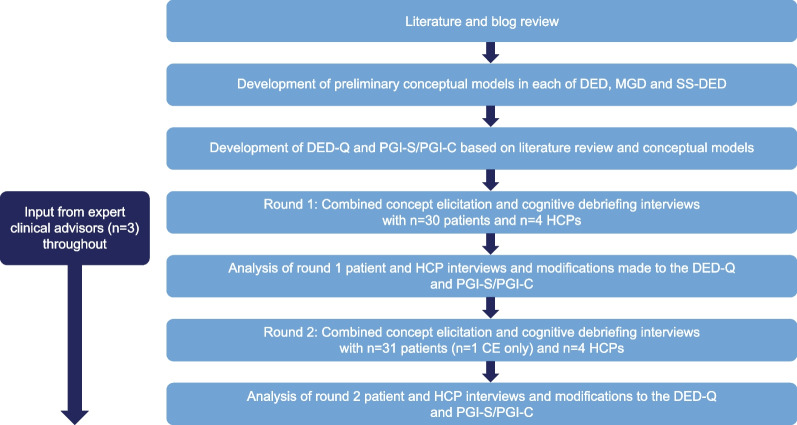
Overview of the study methodology. *CE* concept elicitation, *DED* dry eye disease, *DED-Q* Dry Eye Disease Questionnaire, *HCP* healthcare professional, *MGD* meibomian gland dysfunction, *PGI-C* Patient Global Impression clinical status, *PGI-S* Patient Global Impression disease severity, *SS-DED* Sjögren’s syndrome dry eye disease

This study was a non-interventional, qualitative, semi-structured interview study involving DED, MGD, and SS-DED participants and specialist healthcare professionals (HCPs) to understand the participant experience of these conditions and to adapt and assess the content validity of the DED-Q and PGI-C/PGI-S. Three expert clinical advisors provided input and guidance at key stages of the research (Fig. 1).

### Initial development of the DED-Q

The DED-Q was developed with the aim of assessing the key symptoms, the ability to perform visual activities, and the impact of the conditions on different aspects of participants’ HRQoL. In the first stage, a targeted qualitative literature and blog review was conducted to gain an initial understanding of the key concepts of importance related to participant experiences of DED, MGD, and SS-DED. A total of nine articles [17, 33–39] and six patient blogs [40–45] were reviewed by the study team. For the patient blogs, two individuals read through the posts and coded any HRQoL concepts patients attributed to DED, MGD, or SS-DED. The findings from this review were used to develop three preliminary conceptual models of the participant experience for each condition (Additional file 1). The first draft of the DED-Q (v1_0) included five modules: Eye dryness severity module (single item), Eye dryness frequency module (single item), Symptom module (10 items), Visual tasking module (13 items), and HRQoL module (5 items). The development of the modules for this initial version of the DED-Q was informed by the three conceptual models (eye dryness severity and frequency modules), and previous research [46] conducted by us in DED and chronic ocular surface pain (visual tasking module, symptom module, and HRQoL module)*.* Prior to any interviews being conducted, modifications were made to the initial version of the DED-Q (v1_0) and PGI items (v1_0) based on input from three expert clinical advisors.

### Study participants

Patients were identified by a recruitment agency in the US (i.e., MedQuest) who worked with HCPs to recruit 61 US study participants with a medically confirmed primary diagnosis of DED, MGD, or SS-DED and who were experiencing ocular symptoms due to the condition (e.g., eye dryness, discomfort) in at least one eye. Participants were excluded if they had dry eye symptoms and/or ocular discomfort resulting from refractive surgery in the past year, an ocular infection, or other temporary or intermittent factors causing dry eye symptoms. The detailed eligibility criteria are presented in Additional file 2. The eligibility of the participants was confirmed through documentation in a case report form, and interviews were scheduled after completion of the informed consent form. Interviews were also conducted with eight HCPs (n = 1 Australia, n = 2 Canada, n = 1 France, n = 1 Germany, n = 1 Italy, n = 1 Japan, and n = 1 US) with experience in treating DED, MGD, and SS-DED to ensure that the DED-Q included all clinically relevant and important concepts.

### Interview procedure

The participant interviews, completed via videoconferencing software, were approximately 75 min in length and comprised a 5-min introduction, approximately 25 min of concept elicitation (CE), followed by 45 min of cognitive debriefing (CD).

Interviews were conducted in two rounds (Round 1: n = 30, n = 10 for each condition; Round 2: n = 31, n = 10 for MGD and SS-DED, n = 11 for DED—one participant participated in the CE interview only) to allow for modifications and subsequent testing of the DED-Q between rounds. The aim of the CE portion of the interview was to elicit key concepts of relevance to the participants through a series of broad, open-ended questions designed to elicit spontaneous comments regarding the symptoms and HRQoL impacts of DED, MGD, or SS-DED. Focused probes were then used to explore the topics of interest that emerged from the open-ended questioning. Following the CE portion, the DED-Q (including all modules) and the PGI-C/PGI-S were evaluated with the participants through CD or cognitive interviewing [47]. A “think aloud” approach was used wherein participants were asked to speak their thoughts aloud as they completed each item. The interviewer then asked the participant detailed questions about the definitions/meanings, understanding/clarity, and relevance of each of the instrument items, response options, and recall period.

Interviews with the eight HCPs were similarly designed to first elicit spontaneous comments regarding the symptoms and impacts of DED, MGD, and SS-DED that participants experience because of their condition. In the CD portion, all HCPs were asked to provide feedback on the instructions, items, and response options for each question of the DED-Q in the context of perceived relevance to participants.

### Expert input

Input was sought from three expert clinical advisors (n = 1 US, n = 1 France, n = 1 Greece) to ensure that a clinical perspective of interview findings was obtained, along with gaining feedback regarding the feasibility and appropriateness of using the DED-Q to assess dry eye symptoms and impacts associated with DED, MGD, and SS-DED in future clinical studies. All three expert clinical advisors were ophthalmologists with expertise in at least one of the ocular conditions of interest.

### Data analysis

All interviews were audio-recorded and transcribed verbatim, with identifiable information redacted to anonymize participants. Qualitative analysis was conducted using ATLAS.ti, a computer-assisted software package designed to facilitate storage, coding, and analysis of qualitative data.

The CE portion of the interview transcripts was analyzed using thematic analysis [48, 49]. Participant quotes pertaining to the symptoms and impacts of DED, MGD, or SS-DED were assigned corresponding concept codes in accordance with an agreed coding scheme. A deductive-inductive approach was used to identify themes, whereby codes were applied both deductively (based on prior knowledge) and inductively (emerging from data). Comparisons were also made to identify any differences in the concepts reported between the three conditions. Five members of the study team coded the transcripts across both interview stages. Coding was an iterative process that relied on constant and open communication between the coders throughout the coding process to ensure consistency across transcripts. The concepts identified by each coder were compared and discussed and the coded transcripts were updated to ensure all relevant concepts had been consistency captured across all data sources. Concept saturation [50, 51] was evaluated for the CE participant data to ensure that data collection was exhaustive and that all concepts had been fully explored. The CE findings, as well as input from the expert clinical advisors, were used to update the conceptual models and create a combined single conceptual model to provide a holistic overview of the participant experience of the conditions.

For the CD portion of the transcripts, dichotomous codes were assigned to each item, instruction, response option(s), and recall period to indicate whether it was understood and relevant. The suggested changes to the DED-Q were also coded.

### Ethical considerations and data privacy

The study was approved and overseen by the Western Copernicus Group Independent Review Board (WCG IRB), a centralized ethics review committee in the US (reference: 20204523).

## Results

### Sample characteristics

#### Participant demographics and clinical characteristics

The mean age of the total sample was 50 years (range: 21–80 years). Approximately two-thirds of the participants were female (n = 39/61; 63.9%), with a representation of a range of races: Caucasian (n = 27/61; 44.3%), Black/African American (n = 20/61; 32.8%), Hispanic (n = 7/61; 11.5%), and Asian (n = 4/61; 6.6%). Of the total sample, approximately half of the participants, as rated by the recruiting clinician, were considered to have moderate DED, MGD, or SS-DED (n = 32/61; 52.5%); 15 participants were rated as having severe DED, MGD, or SS-DED (n = 15/61; 24.6%); and 14 participants were rated as having mild DED, MGD, or SS-DED (n = 14/61; 23%). Additional participant demographics and clinical information are detailed in Table 1.

**Table 1:** Participant demographic and clinical characteristics

	DED (n = 21)*	MGD (n = 20)	SS-DED (n = 20)	Total sample (n = 61)
Age (average, range)	47 (21–74)	49 (20–76)	54.5 (29–80)	50.2 (21–80)
Gender (n, %)				
Female	15 (71.4)	15 (75)	9 (45)	39 (63.9)
Male	6 (28.6)	5 (25)	11 (55)	22 (36.1)
Race (n, %)				
White/Caucasian	7 (33.3)	8 (40)	12 (60)	27 (44.2)
Black/African American	10 (47.6)	7 (35)	3 (15)	20 (32.7)
Asian	0 (0)	2 (10)	2 (10)	4 (6.7)
Hispanic/Latino	3 (14.3)	2 (10)	2 (10)	7 (11.5)
Mexican	1 (4.8)	1 (5)	0 (0)	2 (3.3)
Other (not specified)	0 (0)	0 (0)	1 (5)	1 (1.6)
Ethnicity (n, %)				
Non-Hispanic or Latino	15 (71.4)	13 (65)	13 (65)	41 (67.2)
Hispanic or Latino	6 (28.6)	7 (35)	7 (35)	20 (32.8)
Level of education (n, %)				
Some high school	0 (0)	1 (5)	1 (5)	2 (3.3)
High school diploma or GED	9 (42.8)	8 (40)	6 (30)	23 (37.7)
Some years of college	5 (23.8)	3 (15)	3 (15)	11 (18)
Certificate program	1 (4.8)	0 (0)	1 (5)	2 (3.3)
University/College degree (2 or 4 years)	6 (28.6)	6 (30)	3 (15)	15 (24.6)
Graduate or professional degree	0 (0)	2 (10)	6 (30)	8 (13.1)
Severity of eye condition (n, %)				
Mild	3 (14.3)	6 (30)	5 (25)	14 (23)
Moderate	12 (57.1)	10 (50)	10 (50)	32 (52.4)
Severe	6 (28.6)	4 (20)	5 (25)	15 (24.6)

*One DED participant (n = 1) participated in the CE portion of the interview only

*DED* dry eye disease, *DED-Q* Dry Eye Disease Questionnaire, *HRQoL* health-related quality of life, *MGD* meibomian gland dysfunction, *SS-DED* Sjögren’s syndrome dry eye disease, *GED* general educational diploma

#### HCP demographics

The eight HCPs who were interviewed were recruited from Australia (n = 1), Canada (n = 2), France (n = 1), Germany (n = 1), Italy (n = 1), Japan (n = 1), and the US (n = 1) and were working as ophthalmologists (n = 4) or optometrists (n = 4). All HCPs had more than 10 years of experience in their current role and six out of eight HCPs reported treating 10 to 500 individuals with DED, MGD and SS-DED on a monthly basis. (Additional file 2).

### HCP interview CE findings

A total of 25 symptoms of DED, MGD, and SS-DED were discussed by the eight HCPs. The most frequently mentioned symptoms by the HCPs as relevant to the participants across the three conditions included foreign body sensation (DED: n = 8/8, 100%; MGD: n = 8/8, 100%; SS-DED: n = 7/7, 100%), burning sensation (DED: n = 7/8, 87.5%; MGD: n = 7/8, 87.5%; SS-DED: n = 6/7, 85.7%), eyes feeling scratched (DED: n = 7/8, 87.5%; MGD: n = 7/8, 87.5%; SS-DED: n = 6/7, 85.7%), and eye pain/soreness (DED: n = 7/8, 87.5%; MGD: n = 5/8, 62.5%; SS-DED: n = 7/7, 100%). Symptoms less frequently reported for SS-DED compared to DED or MGD included eye irritation, eye itching, eyelid redness, and watery eyes/tearing.

The HCPs described five domains of HRQoL that DED, MGD, and SS-DED participants report to be impacted. The most frequently reported impacts included impacts on reading (DED: n = 7/8, 87.5%; MGD: n = 7/8, 87.5%; SS-DED: n = 6/7, 85.7%) and the use of digital devices (DED: n = 8/8, 100%; MGD: n = 7/8, 97.5%; SS-DED: n = 5/7, 71.4%). The HCPs also mentioned that DED (n = 8/8, 100%), MGD (n = 7/8, 87.5%), and SS-DED (n = 6/7, 85.7%) participants often expressed sadness or depression due to their symptoms. While most HCPs reported that DED (n = 5/8, 62.5%) and SS-DED (n = 5/7, 71.4%) participants experienced impact on driving, fewer HCPs mentioned impact on driving in MGD participants (n = 3/8, 37.5%).

### Participant interview CE findings on symptoms

**Fig. 2:**
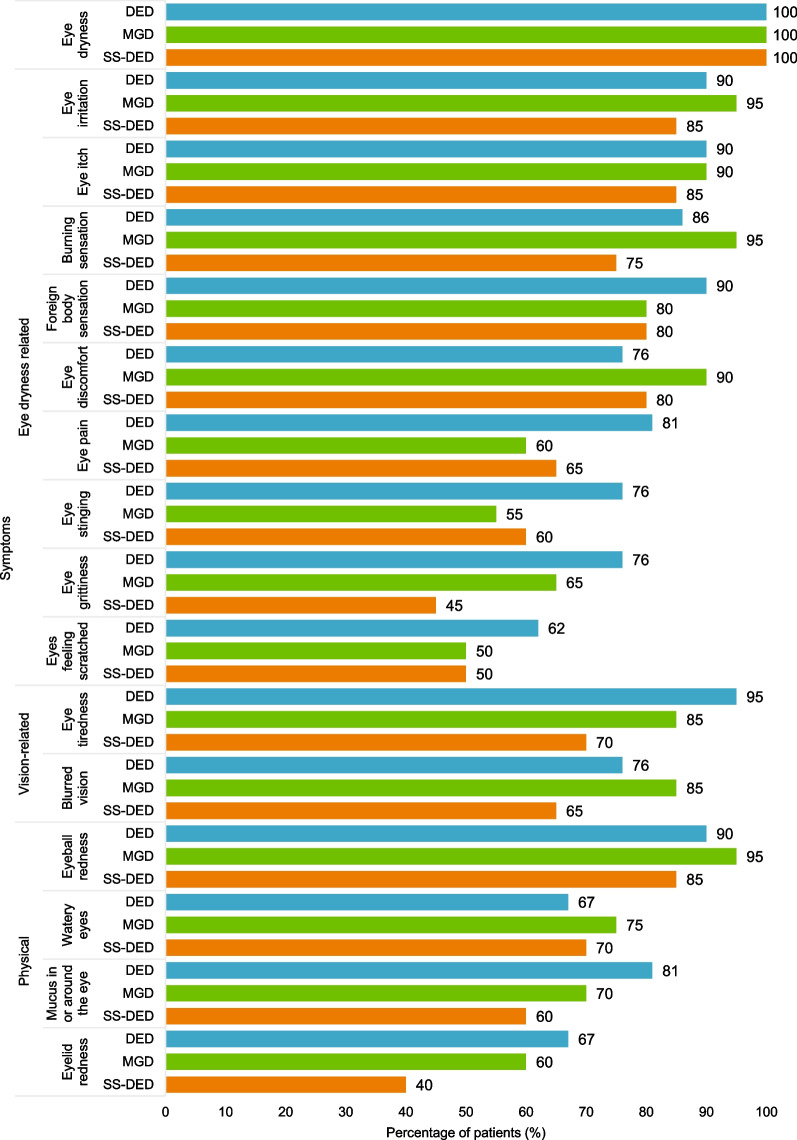
Overview of symptoms reported by more than three patients with DED, MGD, and SS-DED. *DED* dry eye disease, *MGD* meibomian gland dysfunction, *SS-DED* Sjögren’s syndrome dry eye disease

A total of 29 symptoms were reported by the participants across the three conditions, with 16 symptoms (Fig. 2) being relevant to all three conditions (i.e., reported by at least three participants for each condition). While all the participants reported experiencing eye dryness (n = 61/61; 100%) as their key symptom, the most frequently reported eye dryness–related symptoms across the three conditions were eye irritation (n = 55/61; 90%) and eye itch (n = 54/61; 89%). In terms of vision-related symptoms, eye tiredness was reported by a larger proportion of participants with DED (n = 20/21; 95%) and MGD (n = 17/20; 85%) than by participants with SS-DED (n = 14/20; 70%). Blurred vision was reported by most participants with DED (n = 16/21; 76%) and MGD (n = 17/20; 85%) and by a slightly smaller proportion of SS-DED participants (n = 13/20; 65%). The most frequently reported physical symptom across the three conditions was eyeball redness (n = 55/61; 90%).

Across all three conditions, the most bothersome symptoms reported were eye dryness (n = 10 [DED: n = 2; MGD: n = 4; SS-DED: n = 4]), burning sensation (n = 10 [DED: n = 5; MGD: n = 3; SS-DED: n = 2]), and eye irritation (n = 10 [DED: n = 6; MGD: n = 2; SS-DED: n = 2]). However, the most bothersome symptoms varied between conditions, with a greater proportion of DED participants reporting eye irritation, burning sensation, eye grittiness, foreign body sensation, and eye itching as most bothersome; a greater proportion of MGD participants reporting blurred vision, watery eyes, and eye pain as most bothersome; and a greater proportion of SS-DED participants reporting eye dryness, eyeball redness, and eye tiredness as most bothersome.

Symptom subgroup analysis by condition severity showed that a greater proportion of participants in the severe subgroup reported experiencing eye irritation (n = 15/15; 100%), foreign body sensation (n = 15/15; 100%), eye itching (n = 14/15; 93.3%), eye tiredness (n = 14/15; 93.3%), blurred vision (n = 14/15; 93.3%), mucus in or around the eye (n = 12/15; 80%), eye stinging (n = 11/15; 73%), and eye pain (n = 11/15; 73.3%) compared with those in the mild and moderate subgroups. Participants also noted similarities and overlap among the different symptoms. The most frequently reported overlap in symptoms was between foreign body sensation and eye grittiness (n = 11/47; 23.4%), eye irritation and eye dryness (n = 11/47; 23.4%), and eye stinging and burning sensation (n = 11/47; 23.4%).

### Participant interview CE findings on impacts

**Fig. 3:**
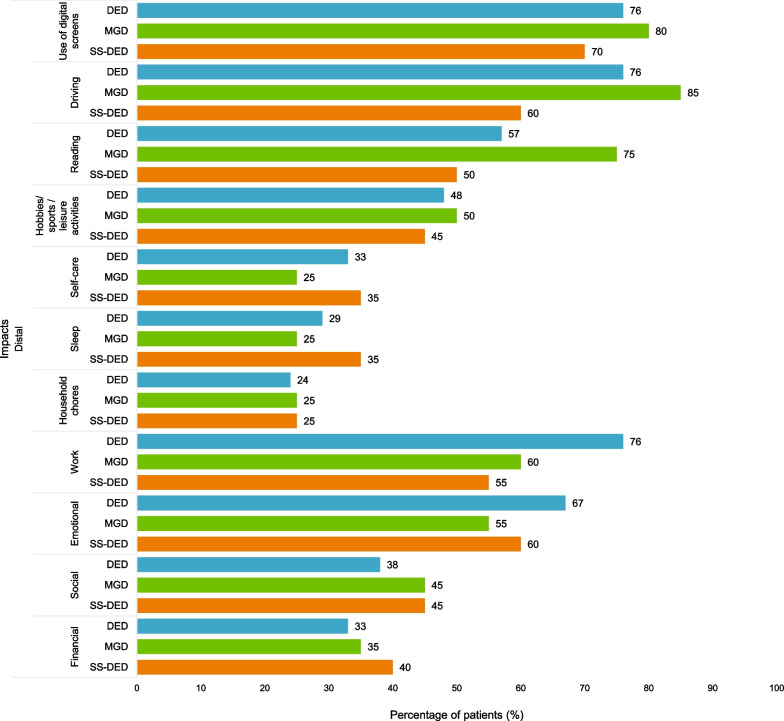
Overview of HRQoL impacts reported by more than three patients with DED, MGD, and SS-DED. *DED* dry eye disease, *HRQoL* health-related quality of life, *MGD* meibomian gland dysfunction, *SS-DED* Sjögren’s syndrome dry eye disease

A total of 14 impacts were reported, of which 11 (Fig. 3) were relevant for participants across the three conditions (i.e., reported by at least three participants for each condition). These impacts were grouped according to whether they were considered proximal or distal to DED, MGD, or SS-DED.

Across the three conditions, the most frequently mentioned impacts were on using digital screens (n = 46/61; 75.4%), driving (n = 45/61; 73.8%), working (n = 39/61; 63.9%), reading (n = 37/61; 60.7%), and emotional well-being (n = 37/61; 60.7%). In contrast, fewer participants reported an impact on their self-care activities (n = 19/61; 31.1%), sleep (n = 18/61; 29.5%), and ability to perform household chores (n = 15/61; 24.6%).

Participants in the severe subgroup experienced greater impact on their use of digital screens (n = 14/15; 93.3%); social functioning (n = 14/15; 93.3%); work (13/15; 86.6%); driving (n = 12/15; 80%); emotional well-being (n = 12/15; 80%); reading (n = 11/15; 73.3%); hobbies, sports, leisure activities (n = 8/15; 53.3%); and sleep (n = 5/15; 33.3%) than those in the mild and moderate subgroups. Overall, much similarity was noted in the way DED, MGD, and SS-DED participants were impacted by their condition.

### Conceptual model

**Fig. 4:**
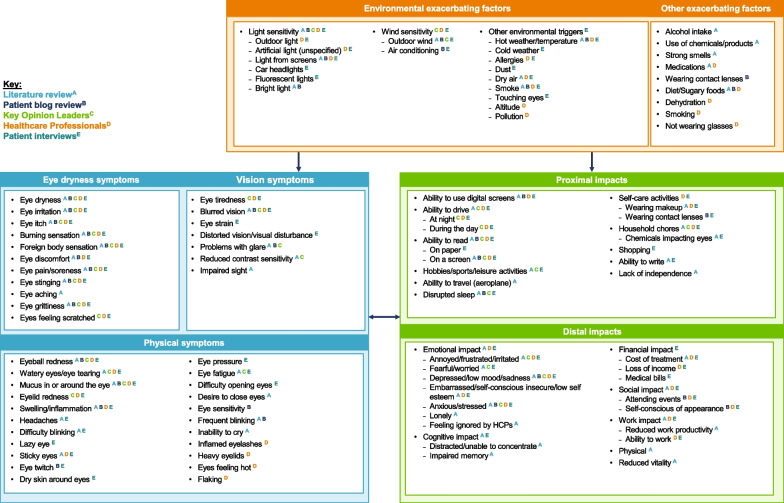
Combined conceptual model with all sources of information. *HCP* healthcare professional

Evidence generated from each round of participant and HCP CE interviews was used to iteratively update the preliminary conceptual model. Figure 4 shows the final version of the conceptual model (with all sources of information).

### Conceptual saturation

Findings from the qualitative analysis suggest that conceptual saturation was achieved for all important symptoms and impacts of DED, MGD, and SS-DED, confirming the adequacy of the study sample size. At the total sample, conceptual saturation analysis found that all concepts were reported spontaneously in the first three sets of patient interviews, except for distorted vision (n = 1) and lazy eye (n = 1). While these symptoms may overlap with other physical or vision-related symptoms, each was only mentioned by one participant, suggesting they may not be related to DED, MGD, or SS-DED, or are at least not considered proximal symptoms. As such, conceptual saturation of the proximal symptoms of DED, MGD, and SS-DED was achieved in the conduct of the first 20 interviews.

At the individual condition level, conceptual saturation was achieved for both MGD and SS-DED samples, with no new concept-relevant information emerging in the final set of interviews, apart from lazy eye (MGD, n = 1) and eyelid redness (SS-DED, n = 1). For the DED sample, three symptom concepts emerged in the final set of interviews: headaches (n = 1), constant blinking (n = 1), and distorted vision (n = 1). Due to the nature of these symptoms, it is likely they could be physiological responses rather than proximal symptoms and therefore evidence suggests that saturation was also achieved for the DED sample. Conceptual saturation of the proximal symptoms of DED, MGD, and SS-DED was achieved in the conduct of the first 10, 12 and 19 interviews respectively.

### Revisions to DED-Q (v1_0) based on the expert clinical advisor input

Recommendations were made by the expert clinical advisors (n = 3) to include four additional modules: dry eye disease symptom severity and frequency modules, blurred vision module, and environmental triggers module. Furthermore, three additional items were added to the symptoms module (i.e., eye redness, eyelid redness, and watery eyes) and five items were removed from the visual tasking module that were deemed less relevant to the experience of DED, MGD, and/or SS-DED.

Modifications were also made to revise the instructions, item stems, item wordings, response option anchors, and examples to improve the comprehension and relevance in DED, MGD, and SS-DED. To maintain consistency in item wording, modifications were also made to the PGI-S (v1_0) and PGI-C (v1_0) to reflect changes to the DED-Q (v1_0).

This updated version, DED-Q (v2_0) used for debriefing in round 1 interviews consisted of the following nine modules:

1. Eye dryness severity module (a single item assessing the severity of participants’ eye dryness right now).
2. Eye dryness frequency module (a single item assessing the frequency of participants’ eye dryness in the past 24 h).
3. Dry eye disease symptom severity module (a single item assessing the severity of participants’ dry eye disease symptoms right now).
4. Dry eye disease symptom frequency module (a single item assessing the frequency of participants’ DED symptoms in the past 24 h).
5. Symptom module (13 items assessing the severity of participants’ eye dryness and associated symptoms).
6. Blurred vision module (2 items assessing the severity of participants’ blurred vision between blinks).
7. Environmental triggers module (2 items assessing the severity of participants’ light and wind sensitivity).
8. Visual tasking module (8 items assessing the impact of DED, MGD, and SS-DED on visual functioning in past 7 days).
9. HRQoL module (5 items assessing the impact of DED, MGD, and SS-DED on sleep and emotional functioning in past 7 days).

The recall periods employed in the DED-Q (v2_0) were developed to capture fluctuating symptom experiences while also accounting for the chronic nature of the conditions. Specifically, the recall period of the eye dryness severity module was revised to “right now” from “the past 4-h” to capture variations in eye dryness and dry eye–related symptom severity at specific points throughout the day. The visual tasking and HRQoL modules employed a 7-day recall, as the impact concepts were more likely to be stable over several days.

### CD findings across DED, MGD, and SS-DED: Round 1

The DED-Q (v2_0), PGI-S (v2_0), and PGI-C (v2_0) were debriefed in Round 1 qualitative interviews with HCPs (n = 4) and participants (n = 30; 10 in each condition) (the content of each of these instrument versions listed in detail can be found in the Additional file 3.). Most HCPs indicated that symptom and impact concepts would be relevant for assessing the patient experience in the populations of interest. Items and response options were well understood by almost all participants, and concepts were largely considered relevant to most participants’ experiences. However, items assessing eye dryness in DED-Q, PGI-S, and PGI-C were misinterpreted by some participants who did not specifically consider their eye dryness when responding to this item, and instead referred to their dry eye disease symptoms more generally when providing their interpretation.

Based on the findings from these interviews, modifications were made to revise the item wording of the Eye dryness severity and frequency modules of DED-Q, PGI-S, and PGI-C to improve participants’ comprehension and encourage participants to think of only eye dryness when providing an answer to the item. These modifications created updated versions of DED-Q (v3_0), PGI-S (v3_0), and PGI-C (v3_0).

Further input was obtained from the three expert clinical advisors on DED-Q v3_0 (forming DED-Q v4_0) who recommended removing the Environmental triggers module as it was determined the concepts of light and wind sensitivity would be difficult to interpret in clinical trials because the changes in scores could be as much due to changes in environmental condition as to changes in symptoms. The instructions of the visual tasking modules were further updated to include examples of possible adjustments that participants might make to help them think more specifically about problems due to their dry eye disease instead of other vision problems they might be experiencing. In addition, modifications were made to the symptoms module to more clearly discriminate eyeball redness from eyelid redness.

### CD findings across DED, MGD, and SS-DED: Round 2

The modified versions of the DED-Q (v4_0), PGI-S (v3_0), and PGI-C (v3_0) were debriefed during Round 2 HCP (n = 4) and participant (n = 30; 10 in each condition) interviews. The items were again generally well understood by almost all participants and most concepts were relevant to greater than 75% of the participants (Additional file 4.). The HCPs also indicated that the symptom and impact concepts included in the DED-Q would be relevant to assess the DED, MGD, and SS-DED populations. However, despite modifications to the items assessing eye dryness, some participants still tended to apply a broader interpretation than intended for most of the items, referring to their overall dry eye disease symptoms rather than the sensation of eye dryness only. Given the overlap in these symptoms being apparent in the CE phase as well, this is arguably a reasonable interpretation. Therefore, all items assessing eye dryness were retained without further modification for testing in a larger sample during the initial psychometric evaluation analyses of the DED-Q. Sample quotations illustrating participant understanding for both the interview rounds are listed in Table 2.

**Table 2:** Example quotes illustrating patient understanding of DED-Q modules/items

Module/item	Example quotes illustrating patient understanding (the patient ID code contains information about the patient including their: sex (male [M]/female [F]), age (in years), primary ocular condition (DED/MGD/SS-DED), condition severity (mild [MILD]/moderate [MOD]/severe [SEV]), round of interviews they were interviewed in (Round 1 [R1]/Round 2 [R2]), and the order they were interviewed)
Eye dryness severity module	“…To rate how bad my—the dryness of my eye is at this moment.” (F42-DED-MOD-R1-15)
Eye dryness frequency module	“It’s kind of in the middle and for the last 24 h I feel like, um, maybe approximately 50% of the time my eyes felt dry and I, you know, went to use my drops because of it.” (F35-MGD-MOD-R2-47)
Dry eye disease severity module	“I'm thinking about, uh, itchy, watery eyes, redness, swelling, um, blurry vision. So, um, I would say right now I'm probably about a four.” (M69-SSDED-SEV-R1-07)
Dry eye disease frequency module	“…Um, I think yesterday was a good day. So, uh, I think some of the time… Yesterday, uh, I felt a bit—like a bit of, uh, the grit feeling, but I put eye drops on.” (M27-DED-MOD-R2-42)
Symptom module	
Eye dryness	“Very dry, um, not enough lubrication, you know, not enough, um, yeah, they're not lubricated, so they're very dry, and, um, they cause, they cause blurriness.” (F42-MGD-MOD-R1-21)
Eye pain	“I'd say within the last day it's probably—the, the worst of it has been a, has been a six where it's actual pain in my eyeball.” (F60-SSDED-SEV-R2-35)
Eye irritation	“Um, eye irritation, it would be—past 24 h, it would be, uh, I'd probably say a six.” (F69-DED-SEV-R1-10)
Burning sensation	“Um, you just feel your eyes are hot. I mean I hardly ever feel burning of the eyes, but I know when people say my eyes are burning, they feel hot, itchy, maybe red.” (F69-MGD-SEV-R1-11)
Eye tiredness	“And the past 24 h, I would say, um, a seven…It’s just, uh, basically having like heavy eyes.” (M40-SSDED-MOD-R2-43)
Something in the eye	“…that answer is 0, and actually I have not had—I have had a feeling of like there was something in my eye but that could have been six months ago.” (F71-DED-MOD-R2-38)
Eye itching	“And I would give that a three within the 24 h. And like I said, at certain times my eye will itch, and I will find myself just rubbing, rubbing, rubbing.” (F59-MGD-MILD-R1-28)
Eye grittiness	“I would say two…because when I feel like there's something in my eye, it feels gritty.” (F71-SSDED-MOD-R1-26)
Mucus in or around the eye	“Well, I would say I do get that around—uh, I'd put a six, I guess. But, um, I do get that mostly in the mornings, so I put a six. It is the worst in the mornings in a 24-h period.” (F71-DED-SEV-R1-06)
Eyes feeling scratched	“Eye feeling scratched. Yes. Because you actually feel like you've injured your eye. Um, in the last 24 h, I have not experienced that. I would give it a zero. I have not had that feeling.” (F39-MGD-MOD-R1-02)
Eyelid redness	“No. Zero. I get no, no—from what I can see, I don’t get no, no redness on my eyelid.” (M48-SSDED-MILD-R2-50)
Eyeball redness	“I'm going to give that a four… because yesterday they were red. They were slightly red. They looked a little irritated and tired.” (F57-DED-MOD-R2-60)
Watery eyes	“So, this morning I would give that a three… Uh, I would describe a three as, um, my eye constantly filling up with water and I'm having to use a, a tissue to—excuse me, to use a tissue to wipe my eye or dab at my eye.” (F59-MGD-MILD-R1-28)
Blurred vision module	
Blurred vision severity	“…it's like things are out of focus and, um, it takes—it seems to take some time, five or ten minutes at least for things to come back into focus.” (M68-SSDED-MOD-R2-31)
Blurred vision frequency	“Um, some of the time… Because it’s not constant, it’s just like in between like every couple of hours.” (F48-DED-SEV-R2-49)
Environmental triggers module	
Light sensitivity	“So, 24 h, so, um, I would give that a four because when I go out into the sunlight, my eyes really—they're really sensitive to where it watered up a lot.” (F59-MGD-MILD-R1-28)
Wind sensitivity	“Just any little—you know, a certain breeze will come and hit me in the face, and I have trouble with it.” (F80-SSDED-SEV-R1-05)
Visual tasking module	
Read books	“Sometimes, some of the times… Especially when I read at night, um, my eyes are really, really tired because of the redness, the burning. Sometimes I, I do experience, um, problems reading books.” (F21-DED-MILD-R1-22)
Read on screen	“I would say some of the time…that's a, a big thing for me 'cause I'm always on the screen. So, um, if I don’t change the brightness, I would definitely feel some irritation…The heaviness, um, I guess, you know, like slightly like the burning but the heaviness of your eyes, like the tired feeling of your eyes actually from staring at the screen so long.” (F43-MGD-MILD-R2-48)
Watch TV	“Watch a program on TV. Uh, I can say just a little of the time. It really doesn’t bother me a lot. It's more—now I realize it's more the computer than the TV.” (F50-SSDED-MOD-R1-01)
Household chores	“none of the time really… It don’t affect. So, it don’t affect me none of the time to do household chores or laundry or cleaning. No. It don't bother me.” (F69-DED-SEV-R1-10)
Hobbies/sports/leisure activities	“Some of the time for this one…Uh, well, when I'm at the gym, you know, um, and I'm sweating and, um, I don't know, I get dry eyes. Um, when I'm doing puzzles, uh, same thing, you know, like I get the blurry vision, I have to step away for a bit and then come back to it.” (F42-MGD-MOD-R1-21)
Watch events at distance	“Um, with this one, probably some of the time. And depending on the event, maybe a lot of the time…I would still of course like musical events, but the sporting events, it, uh—um, to me it's not worth watching a sporting event if my eyes are going to get tired.” (F36-SSDED-MOD-R2-32)
Drive during day	“…A lot of the times because I'm looking at the car ahead of me and so I'm zeroing in on something ahead and it seems to be, uh, blurry sometimes… So that is a problem. And I put a lot of the time.” (F71-DED-SEV-R1-06)
Drive at night	“So, in the past seven days, driving at night, I have driven at night and, uh, I don’t really feel any symptoms because there's no sunlight and I do have my windows up. So, I would say none of the time for that really, you know, when I'm driving.” (M20-MGD-MILD-R2-59)
HRQoL module	
Depression	“I was working on a job and I wasn’t able to complete it when I needed to and that, that—I felt depressed about that because it was an important one to me.” (F60-SSDED-SEV-R2-35)
Anxiety	“…I would say, um, a little of the time” “There’s times when I’m anxious to kind of relieve the irritation so that I could continue with my day. So yeah, a little of the time.” (M29-DED-MOD-R2-46)
Frustration	“Frustrated, some of the time…It makes you frustrated because sometimes, uh, nothing really helps. Nothing helps, um, you know, in an extreme manner. Sometimes it's just very frustrating just not knowing what to do.” (F39-MGD-MOD-R1-02)
Worry	“I don’t like to, you know, try to see what's going to happen in the future, but I can't predict it, but, you know, it does make me feel like, you know, oh, well, you know, I feel a little bit worried.” (M48-SSDED-MILD-R2-50)
Sleep	“Um, I'm going to say one to two because just waking up trying to stop them from feeling, um, irritated…” (F44-DED-SEV-R1-13)

*DED* dry eye disease, *DED-Q* Dry Eye Disease Questionnaire, *HRQoL* health-related quality of life, *MGD* meibomian gland dysfunction, *SS-DED* Sjögren’s syndrome dry eye disease, *TV* television

Regarding item relevance, small differences were observed between the conditions, with more MGD patients indicating that eyelid redness, watery eyes, and feelings of anxiety were relevant to their disease experience (compared to DED and SS-DED participants), whereas driving at night was considered less relevant by MGD participants than by DED or SS-DED participants. When examined by severity subgroups, only minor differences in the relevance of concepts were observed between severity levels, with fewer mild participants reporting that eye grittiness and feelings of anxiety and worry were relevant to them and fewer severe participants reporting that difficulty doing household chores was relevant.

### Understanding and relevance of the recall periods across interview rounds

Three recall periods are used in the DED-Q (v3_0) to correspond with the presentation of symptoms described by participants. The eye dryness and dry eye disease modules use a recall period of ‘right now’; the eye dryness frequency, dry eye disease frequency, and symptom module use a recall period of the ‘past 24 h’; and the visual tasking and HRQoL modules use a recall period of the ‘past 7 days’. For most modules in the DED-Q (v3_0), ≥ 80% of participants understood and used the recall period correctly. The recall period for the HRQoL module was least well understood with only 58% of participants demonstrating a clear understanding. Except for ‘watching sports at a distance’ of the visual tasking module, > 50% participants who reported an item to be relevant to their experience also reported experiencing the concept within the recall period.

## Discussion

The current study was conducted to address the unmet need for a PRO measure with evidence of content validity for use in the specific context of DED, MGD, and SS-DED. Given the lack of sufficient published evidence on patient experience in these conditions, qualitative research was undertaken in line with best practices outlined in FDA PRO guidance [23–26] to supplement the initial findings from the literature and patient blog reviews. Specifically, participant experiences of DED, MGD, and SS-DED were explored to generate evidence to support the content validity of the DED-Q, PGI-C, and PGI-S as suitable measures for use across the conditions.

The primary symptoms most reported by participants across both rounds of CE interviews were eye dryness, eye irritation, eye pain, eye itch, burning sensation, and foreign body sensation. Participants reported that DED, MGD, and SS-DED had substantive impacts on several domains of daily life, with the most notable impacts being those on activities of daily living (e.g., using digital screens, driving, reading, and working) and emotional well-being. These findings were largely consistent with concepts identified from the published evidence and what was reported by the HCPs and were recognized as relevant by the three expert clinical advisors [12, 18–20].

The initial version of the DED-Q (v1_0), which was developed based on the findings from literature and blog reviews, included modules assessing DED, MGD, and SS-DED symptoms and associated visual and wider HRQoL impacts. Based on input from the expert clinical advisors, revisions were made to the instructions and items of DED-Q (v1_0) modules along with the addition of the following three modules: dry eye disease symptom severity and frequency module, blurred vision, and environmental triggers, leading to the formation of DED-Q (v2_0).

CD interviews for the updated version conducted with four HCPs indicated that all symptom and impact concepts included in the DED-Q (v2_0) were relevant to DED, MGD, and SS-DED populations. In the first round of participant interviews, most items and response options were well understood, and the concepts were generally considered relevant to participant experiences. However, modifications were required for the wording and response anchors for DED-Q (forming DED-Q [v3_0]) and PGI items which assessed eye dryness to improve participant comprehension by encouraging participants to focus only on eye dryness when selecting a response (rather than focusing on all symptoms). Based on feedback from the three expert clinical advisors on the DED-Q (v3_0), the item wording and instructions were modified further, along with the examples to be more specific to the three conditions of interest. Additionally, the environmental triggers module was removed because it was agreed that the data would be challenging to collect and interpret during the clinical trials. In Round 2 of the HCP and participant interviews, the DED-Q (v4_0) items were again generally well understood and most concepts were found to be relevant to most participants. However, despite modifying the items assessing eye dryness, some participants appeared to still apply a broader interpretation than intended (i.e., focusing on all symptoms rather than eye dryness alone). Given the overlap in symptom concepts identified during the CE phase with both participants and HCPs, this is a reasonable interpretation. Therefore, all items assessing eye dryness were retained without further modification for testing in a larger sample during the initial psychometric evaluation analyses of the DED-Q to ensure that the most important and strongest performing items were retained. Whilst results demonstrated that most participants understood and deemed the recall periods used within the DED-Q appropriate, understanding of the 7-day recall period in the HRQoL module was lower. It is likely that this is a result of the interview design in which participants debriefed all modules of the DED-Q in its entirety. In a real world setting the modules would likely be used independently in line with their associated recall periods, therefore it is anticipated that this would allay any discrepancies identified in this research.

To our knowledge, this is the first qualitative research designed in line with regulatory PRO guidance and involving an active collaboration with experts and specialist HCPs to gain an in-depth understanding of participant experiences of DED, MGD, and SS-DED. The findings support the prioritization of eye dryness and related symptoms for PRO symptom assessment in these three conditions. There is also evidence supporting the importance of measuring visual functioning and impact concepts to fully evaluate the impact of DED, MGD, and SS-DED on participants’ lived experiences. As no major differences were observed between the three conditions, the findings support the use of a single instrument across these three conditions instead of deploying a separate instrument for each. The severity scores/levels of endorsement of different items may vary across the conditions when examined in larger studies. Furthermore, it is possible that during psychometric evaluation different item sets are appropriate for the different conditions. At this point, qualitative evidence suggests that the same items are relevant for use in all three conditions.

It should be recognized that while the study design provided considerable depth of insight and descriptions regarding the participant experience, conclusions must be drawn considering the study limitations that one would typically expect from qualitative research. Specifically, fewer participant with mild and severe disease (as reported by the recruiting clinician) were included in the DED (mild [n = 3], moderate [n = 10], and severe [n = 4]), MGD (mild [n = 6], moderate [n = 10], and severe [n = 4]), and SS-DED (mild [n = 5], moderate [n = 10], and severe [n = 5]) groups. However, given the similarity in symptoms reported by participants, regardless of the disease severity, the relevance and suitability can be inferred with confidence, as saturation was achieved, and no new concepts were identified. Furthermore, due to time constraints and to minimize participant burden, not all modules were debriefed to all participants. However, enough participants were debriefed on each module, and where data were lacking, the CE findings were extrapolated to support evidence of concept relevance.

## Conclusions

This qualitative study followed rigorous and recommended methods with sufficient participant and clinical input for the adaptation of a newly developed PRO measure for its use in DED, MGD, and SS-DED. The two rounds of HCP and participant interviews, along with clinical advisor input, provided an in-depth understanding of the participant experience of living with DED, MGD, and SS-DED. The evidence generated from the interviews contributed to a combined conceptual model of DED, MGD, and SS-DED experiences. The CD findings support the content validity of DED (v4_0) and the two PGI items (v3_0) as three PRO measures appropriate for use in clinical studies to assess the participant experience of DED, MGD, and SS-DED. Additional psychometric analysis studies are needed to further refine these measures and confirm their suitability to support clinical trial endpoints for eventually backing claims of treatment benefit in product labeling.

## Supplementary Information

**Additional file 1.** Preliminary conceptual models of the participant experiences of DED, MGD and SS-DED.

**Additional file 2.** Participants’ eligibility criteria.

**Additional file 3.** Instrument versions: DED-Q, PGI-S, PGI-C.

**Additional file 4.** CD interviews: Participant understanding and relevance findings for the DED-Q modules.

## Data Availability

Data are available from the authors upon reasonable request and with permission of Novartis Pharma AG.

## Abbreviations

CD: Cognitive debriefing
CE: Concept elicitation
DED: Dry eye disease
DED-Q: Dry Eye Disease Questionnaire
DEQ: Dry Eye Questionnaire
DEWS: Dry Eye Workshop
FDA: Food and Drug Administration
HCP: Healthcare professional
HRQoL: Health-related quality of life
IDEEL: Impact of dry eye on everyday life
MGD: Meibomian gland dysfunction
OCI: Ocular comfort index
OSDI: Ocular surface disease instrument
PGI-C: Patient global impression of change
PGI-S: Patient global impression of severity
PRO: Patient-reported outcome
SANDE: Symptom assessment in dry eye
SPEED: Standard patient evaluation of eye dryness questionnaire
SS-DED: Sjögren’s syndrome dry eye disease
TFOS: Tear Film and Ocular Surface Society
US: United States
