# Supplementary material for: Development and content validity assessment of the Dry Eye Disease Questionnaire in patients with dry eye disease, meibomian gland dysfunction, and Sjögren’s syndrome dry eye disease
Source: J Patient Rep Outcomes. 2023 Jul 5;7:64. doi: 10.1186/s41687-023-00608-5 (PMC10323053; doi:10.1186/s41687-023-00608-5)

Fig. 1: Preliminary conceptual model of patient experience: DED


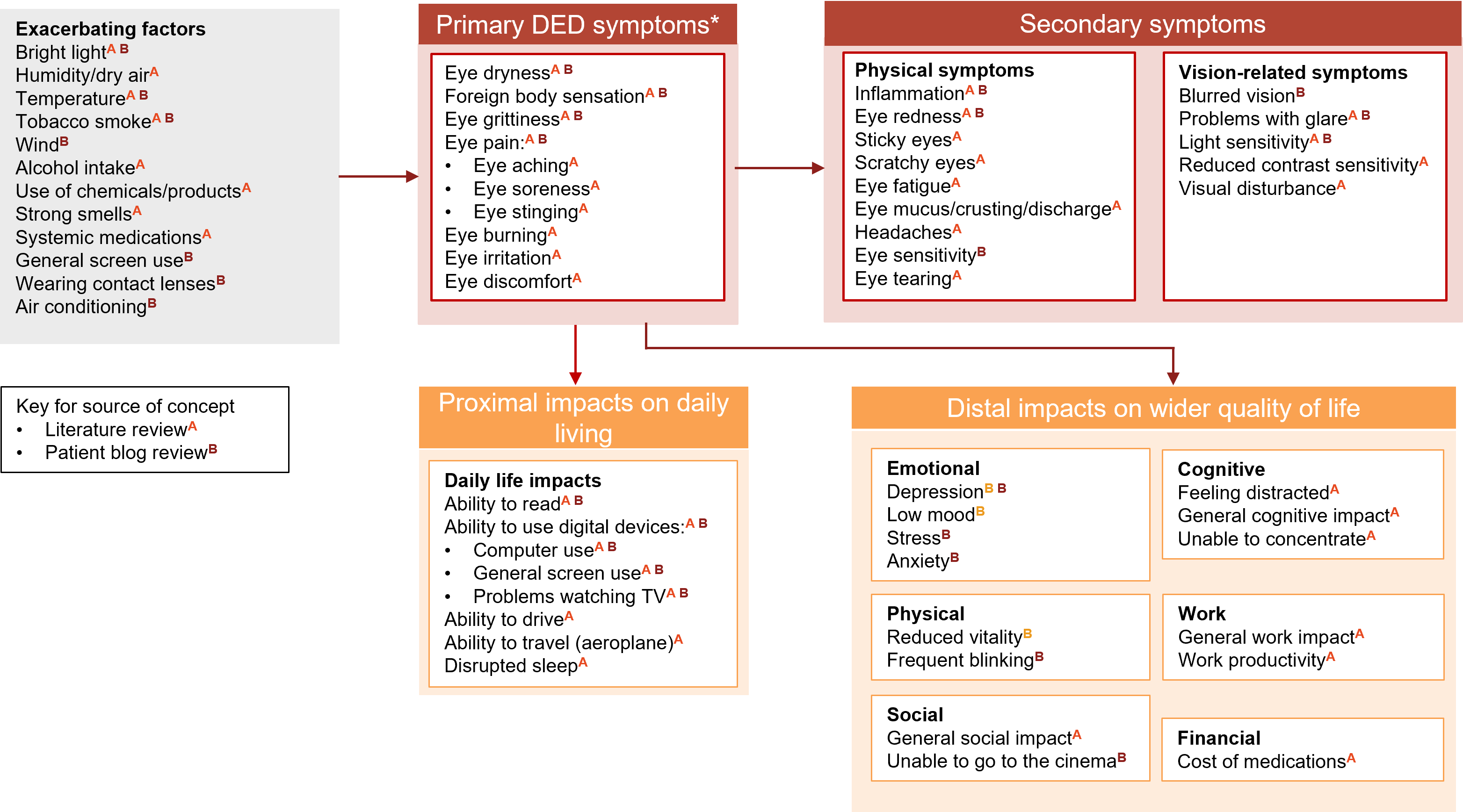


Fig. 2: Preliminary conceptual model of patient experience: MGD


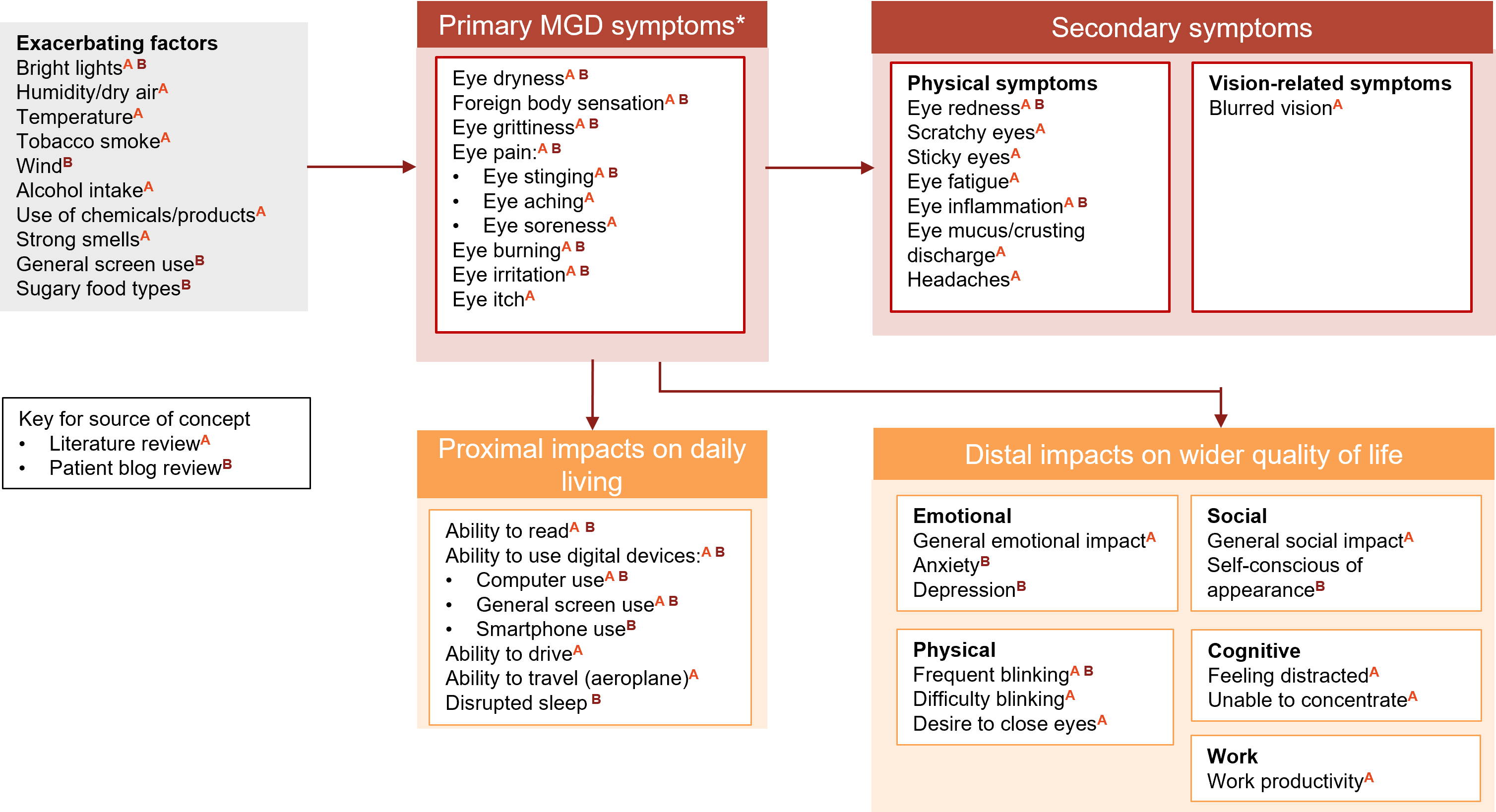


Fig. 3: Preliminary conceptual model of patient experience: SS-DED


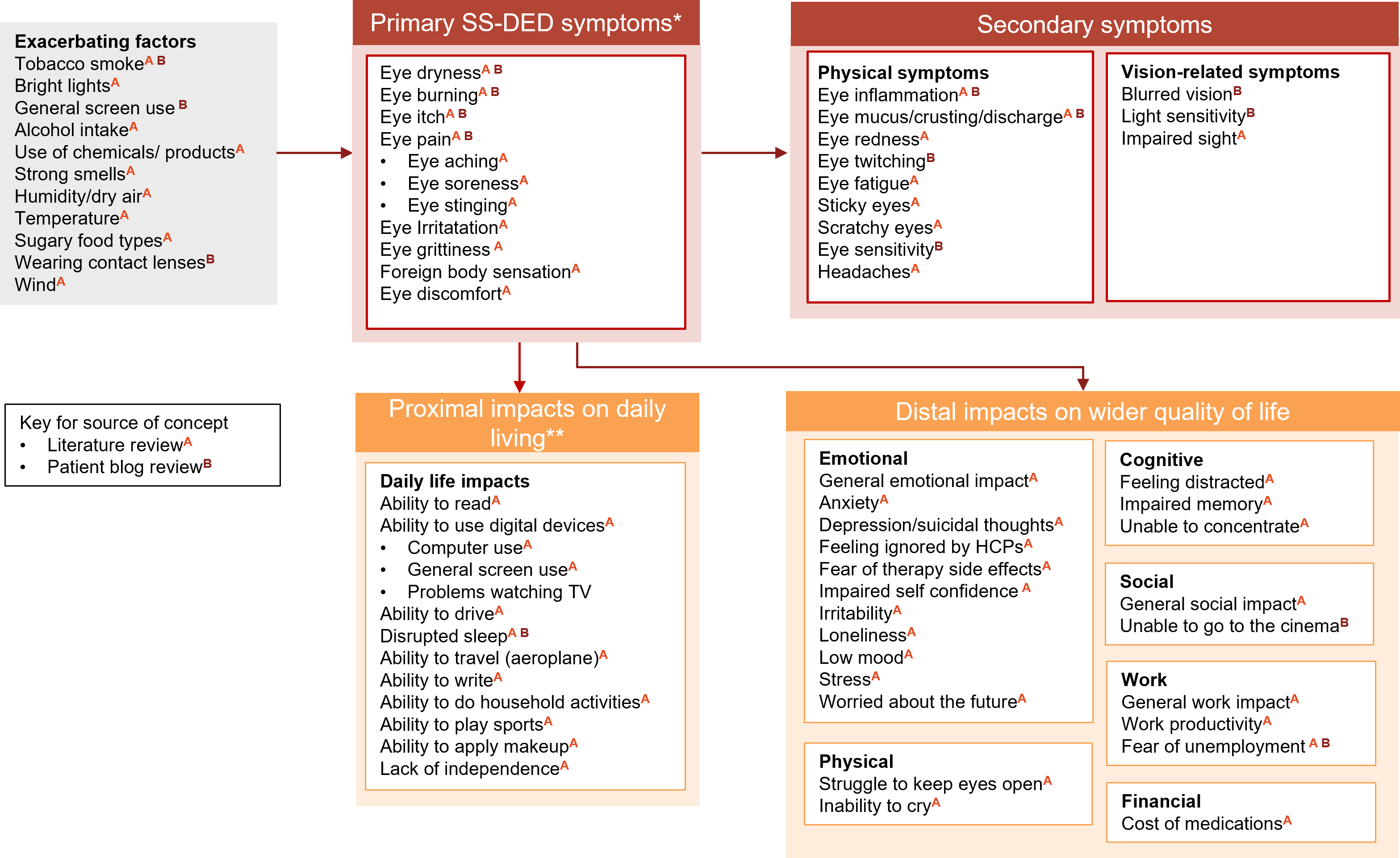

Supplement: Supplementary file 1 — Additional file 1. Preliminary conceptual models of the participant experiences of DED, MGD and SS-DED. [file 41687_2023_608_MOESM1_ESM.docx]
