# Supplementary material for: Development and content validity assessment of the Dry Eye Disease Questionnaire in patients with dry eye disease, meibomian gland dysfunction, and Sjögren’s syndrome dry eye disease
Source: J Patient Rep Outcomes. 2023 Jul 5;7:64. doi: 10.1186/s41687-023-00608-5 (PMC10323053; doi:10.1186/s41687-023-00608-5)
Supplement: Supplementary file 2 — Additional file 2. Participants’ eligibility criteria. [file 41687_2023_608_MOESM2_ESM.docx]

Eligibility criteria:

Patients were eligible to participate in the research if they:

1. Were aged 18 years or older.
2. Had a clinician-confirmed primary diagnosis of DED, MGD, or SS-DED and was currently experiencing ocular symptoms due to the condition (e.g., eye dryness, discomfort) in at least one eye regardless of the treatments they had received.
3. Had an eye exam in the past six months with evidence (signs and symptoms) of their primary diagnosis.
4. Were a fluent speaker, literate, and able to read and write in English language.
5. Were willing and able to provide informed consent and participate.

Patients were excluded from the research if they:

1. Had dry eye symptoms and/or ocular discomfort resulting from refractive surgery in the past year, an ocular infection at the time of screening, or an episodic condition related to environmental conditions, seasonal or geographical factors, or other temporary or intermittent factors.
2. Had active, or history of, ocular allergies during the time of year they were expected to participate.
3. Had used contact lenses in either eye in the past 14 days.
4. Were unable to understand or comprehend the study information.
5. Had any other physical condition, mental condition, or learning difficulty that may have impacted their participation.

| Recruitment quotas  The following patient quotas were implemented to ensure representation of important demographic and clinical subgroups  Table 1. Proposed sampling quotas for each of the DED, MGD and SS-DED subgroups (n=20 in each) | | |
| --- | --- | --- |
| **Proposed study quotas (n=20 in each of DED, MGD and SS-DED)** | | |
| **Patient characteristics** | | **Total** |
| Age | 18-35 | ≥5 |
|  | 36-60 | ≥5 |
|  | 61+ | ≥5 |
| Gender | Male | ≥8 |
|  | Female | ≥8 |
| Educational attainment | Completed high school or less | ≥5 |
|  | Some higher education beyond high school | ≥5 |
| Race | Non-Caucasian | ≥5 |
|  | Caucasian | ≥5 |
| Ethnicity | Hispanic/Latino | ≥5 |
|  | Non-Hispanic/non-Latino | ≥5 |
| Clinician-rated severity of ocular symptoms | Diagnosis of mild MGD, DED or SS-DED (quota for each condition) | 3-5 |
|  | Diagnosis of moderate MGD, DED or SS-DED (quota for each condition) | 6-9 |
|  | Diagnosis of severe MGD, DED or SS-DED (quota for each condition) | 6-10 |
