# Supplementary material for: Development and content validity assessment of the Dry Eye Disease Questionnaire in patients with dry eye disease, meibomian gland dysfunction, and Sjögren’s syndrome dry eye disease
Source: J Patient Rep Outcomes. 2023 Jul 5;7:64. doi: 10.1186/s41687-023-00608-5 (PMC10323053; doi:10.1186/s41687-023-00608-5)
Supplement: Supplementary file 3 — Additional file 3. Instrument versions: DED-Q, PGI-S, PGI-C. [file 41687_2023_608_MOESM3_ESM.docx]

# DED-Q v1_0

**Eye Dryness Severity Module**

| **1. Eye dryness** | Please rate the severity of your eye dryness **in the past 4 hours**:  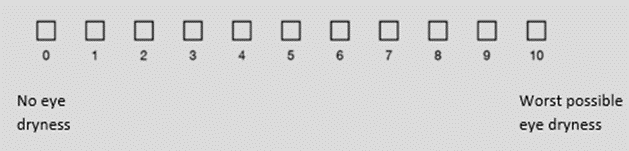 |
| --- | --- |

**Eye Dryness Frequency Module**

| **1. Eye dryness frequency** | How much of the time have you had eye dryness **in the past 24 hours**?   \| 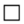 \| 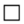 \| 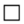 \| 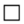 \| 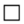 \|  \| \| --- \| --- \| --- \| --- \| --- \| --- \| \| None of the time \| A little of the time \| Some of the time \| A lot of the time \| All of the time \| |
| --- | --- | --- | --- | --- | --- | --- | --- | --- | --- | --- | --- | --- |

**Symptom Module**

Please answer the following questions thinking about each symptom at the time it was **at its worst** over the past 24 hours.

| **1. Eye dryness** | Please rate the severity of your eye dryness **at its worst** in the past 24 hours:  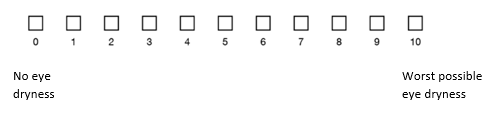 |
| --- | --- |
| **2. Eye pain** | Please rate the severity of your eye pain **at its worst** in the past 24 hours:  **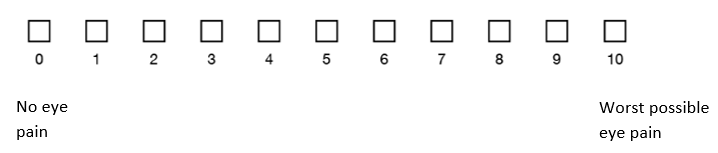** |
| **3. Eye irritation** | Please rate the severity of your eye irritation **at its worst** in the past 24 hours: **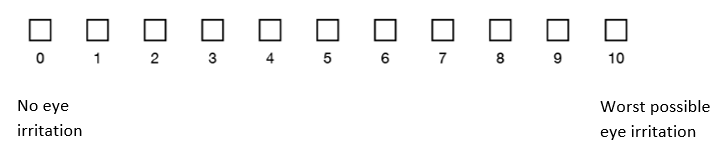** |
| **4. Burning in eye** | Please rate the severity of any burning feelings in your eye(s) **at its worst** in the past 24 hours:  **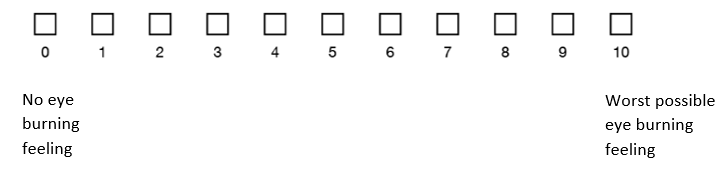** |
| **5. Eye tiredness** | Please rate the severity of your eye tiredness **at its worst** in the past 24 hours:  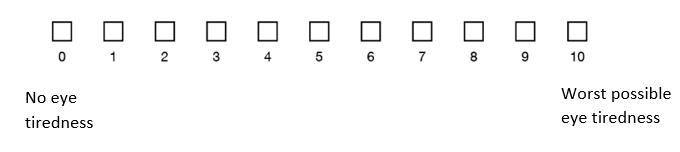 |
| **6. Feeling like there is something in your eye** | Please rate the severity of a feeling that you have something in your eye **at its worst** in the past 24 hours:  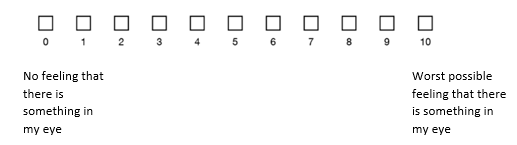 |
| **7. Eye itch** | Please rate the severity of the itch in your eye **at its worst** in the past 24 hours:  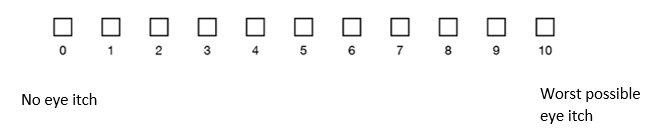 |
| **8. Eye grittiness** | Please rate the severity of a gritty feeling in your eye **at its worst** in the past 24 hours:  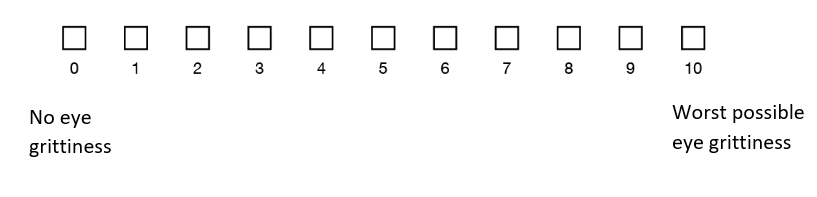 |
| **9. Mucus in or around the eye** | 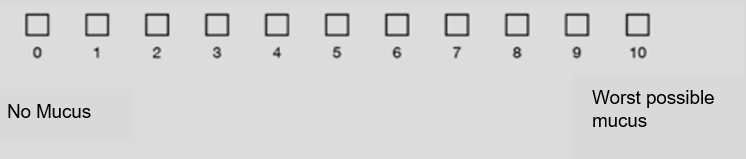Please rate the severity of mucus in or around your eye **at its worst** in the past 24 hours: |
| **10. Eyes feeling scratched** | Please rate the severity of your eyes feeling like they have been scratched by something **at its worst** in the past 24 hours:  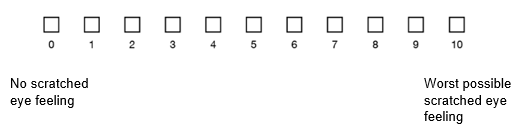 |

**Visual Tasking Module**

The following questions ask about how much of the time your **eye dryness affected or interfered with your ability to do visual activities in the past 7 days.**

Difficulties doing visual activities might include **changing how you did an activity, avoiding an activity, or needing to take a rest from an activity** because of your eye dryness.

Please answer the questions thinking about how much of the time these activities are affected when you **do not make adjustments** (for example, think about reading on your phone **without** increasing the font size).

| Over the past 7 days how much of the time did your eye dryness affect your ability to… | None of the time | A little of the time | Some of the time | A lot of the time | All of the time | I avoided or was completely unable to do this activity due to my eye dryness | Not applicable I did not do this for reasons unrelated to my eye dryness |
| --- | --- | --- | --- | --- | --- | --- | --- |
| 1. Read or type a text message on your cell-phone (without increasing font size)? |  |  |  |  |  |  |  |
| 2. Look in the mirror for example to shave or put your make up on? |  |  |  |  |  |  |  |
| 3. Read on paper for example books, newspapers or magazines? |  |  |  |  |  |  |  |
| 4. Read labels, instructions, ingredients, or prices for example on medicine bottles, food packaging, or receipts? |  |  |  |  |  |  |  |
| 5. Read on a screen for example a computer or tablet (without increasing font size)? |  |  |  |  |  |  |  |
| 6. Watch a program on a screen for example TV or a tablet? |  |  |  |  |  |  |  |
| 7. Work on a computer screen (without increasing font size)? |  |  |  |  |  |  |  |
| 8. Carry out household chores for example cleaning or laundry? |  |  |  |  |  |  |  |
| 9. Carry out your usual leisure activities or hobbies for example crafts, painting, playing cards? |  |  |  |  |  |  |  |
| 10. Watch events at a distance for example a show or sporting event? |  |  |  |  |  |  |  |
| 11. Locate items in a store when shopping? |  |  |  |  |  |  |  |
| 12. Drive during the day? |  |  |  |  |  |  |  |
| 13. Drive at night? |  |  |  |  |  |  |  |

**Health-Related Quality of Life Module**

The following questions ask about **ways your eye dryness may have affected you in the past 7 days.**

For each question, please choose the answer which describes how much of the time you were affected **because of your eye dryness in the past 7 days**.

| Over the past 7 days… | | None of the time | A little of the time | Some of the time | A lot of the time | All of the time |
| --- | --- | --- | --- | --- | --- | --- |
| 1 | How much of the time did you feel low or depressed? |  |  |  |  |  |
| 2 | How much of the time did you feel anxious? |  |  |  |  |  |
| 3 | How much of the time did you feel frustrated? |  |  |  |  |  |
| 4 | How much of the time did you feel worried? |  |  |  |  |  |
| 5 | How many nights did your eye dryness affect your sleep? | - 0 nights - 1-2 nights - 3-4 nights - 5-6 nights - Every night | | | | |

# PGI-S and PGI-C v1_0

**Global Impression of Severity**

**1.** Please choose the response below that best describes the overall severity of your **eye dryness** over the **past 7 days.**

□ Severe

□ Moderate

□ Mild

□ None

**2.** Please choose the response below that best describes the overall severity of your **limitations in carrying out visual activities (e.g. reading, watching the TV)** **due to eye dryness** over the **past 7 days.**

□ Severe

□ Moderate

□ Mild

□ None

**Global Impression of Change**

**1.** Please choose the response below that best describes the overall change in your **eye dryness** compared to when you started taking the study treatment.

□ Much better

□ A little better

□ No change

□ A little worse

□ Much worse

**2.** Please choose the response below that best describes the overall change in your **limitations in carrying out visual activities (e.g. reading, watching the TV) due to eye dryness** compared to when you started taking the study treatment (select one response).

□ Much better

□ A little better

□ No change

□ A little worse

□ Much worse

# DED-Q v2_0 debriefed in round 1 HCP and patient interviews

**Eye Dryness Severity Module**

| **1. Eye dryness severity** | Please rate the severity of your eye dryness **right now**:  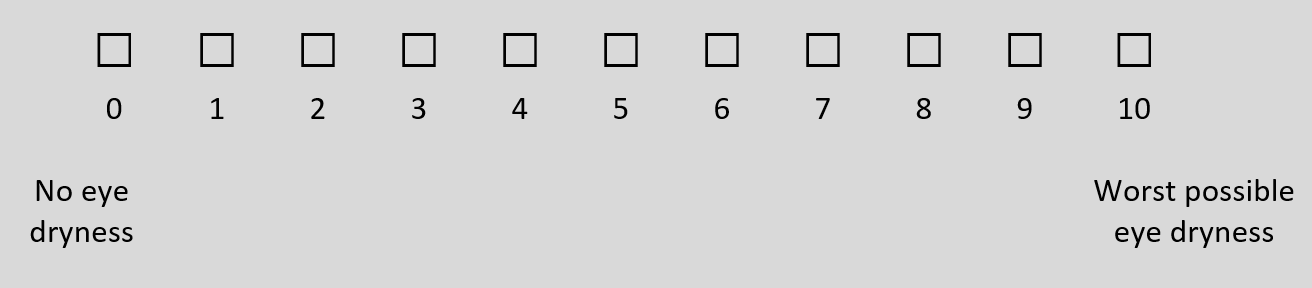 |
| --- | --- |

**Eye Dryness Frequency Module**

| **1. Eye dryness frequency** | How much of the time have you had eye dryness **in the past 24 hours**?   \| 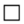 \| 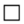 \| 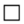 \| 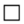 \| 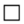 \|  \| \| --- \| --- \| --- \| --- \| --- \| --- \| \| None of the time \| A little of the time \| Some of the time \| A lot of the time \| All of the time \| |
| --- | --- | --- | --- | --- | --- | --- | --- | --- | --- | --- | --- | --- |

**Dry Eye Disease Symptom Severity Module**

| **1. Dry eye disease symptom severity** | Please rate the severity of your dry eye disease symptoms **right now**:  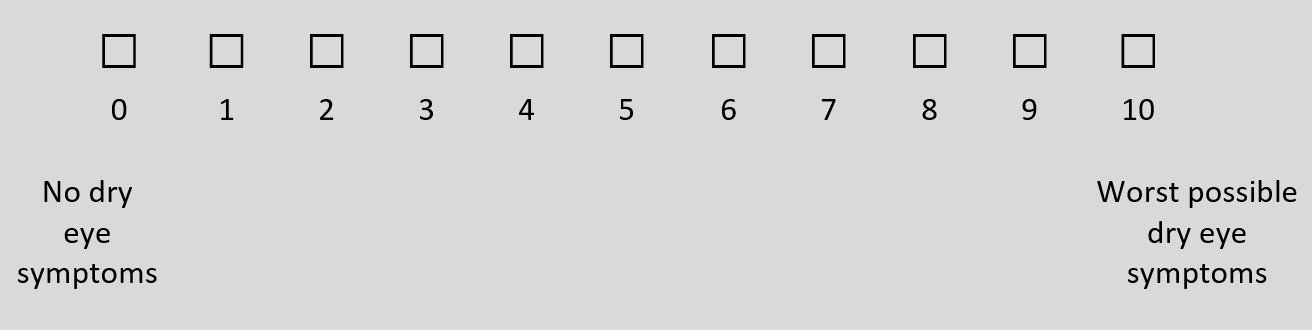 |
| --- | --- |

**Dry Eye Disease Symptom Frequency Module**

| **1. Dry eye disease symptom frequency** | How much of the time have you had dry eye disease symptoms **in the past 24 hours**?   \| 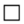 \| 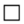 \| 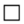 \| 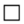 \| 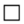 \|  \| \| --- \| --- \| --- \| --- \| --- \| --- \| \| None of the time \| A little of the time \| Some of the time \| A lot of the time \| All of the time \| |
| --- | --- | --- | --- | --- | --- | --- | --- | --- | --- | --- | --- | --- |

**Symptom Module**

Please answer the following questions thinking about each symptom at the time it was **at its worst** over the past 24 hours.

| **1. Eye dryness** | Please rate the severity of your eye dryness **at its worst** in the past 24 hours:  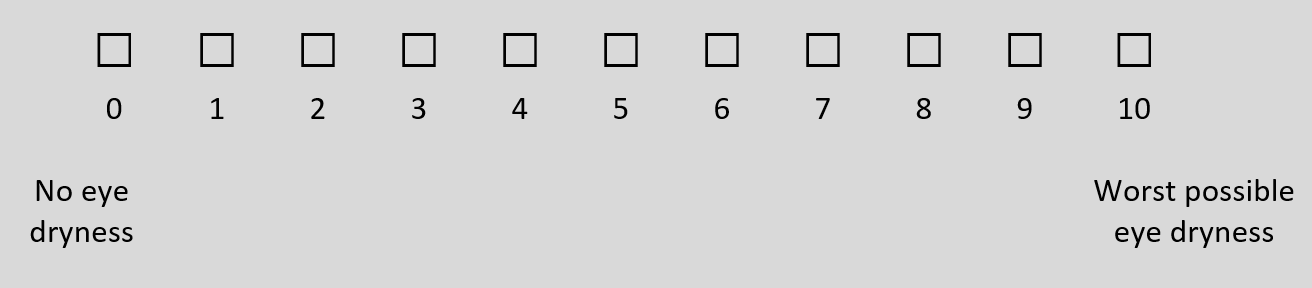 |
| --- | --- |
| **2. Eye pain** | Please rate the severity of your eye pain **at its worst** in the past 24 hours:  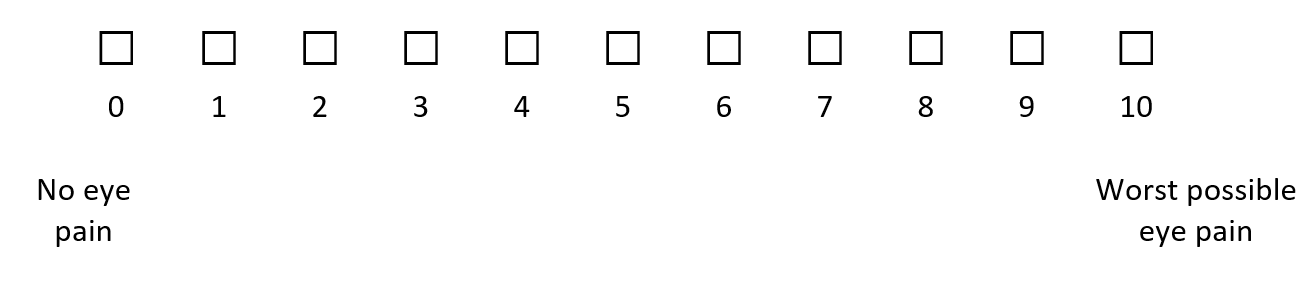 |
| **3. Eye irritation** | Please rate the severity of your eye irritation **at its worst** in the past 24 hours:  **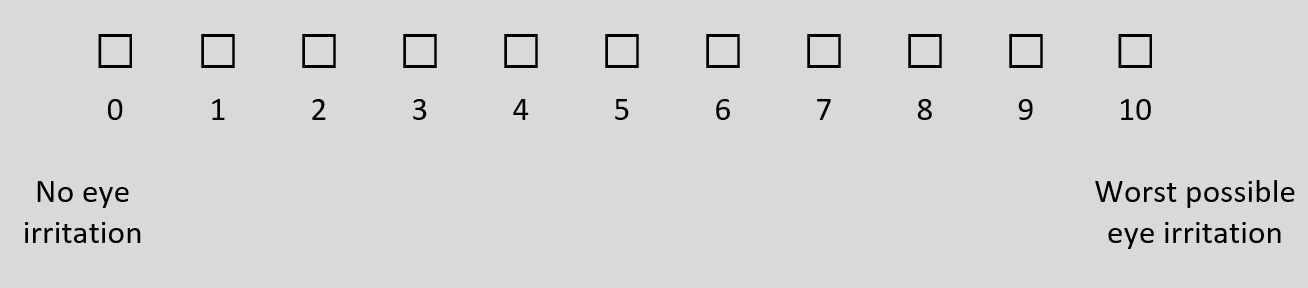** |
| **4. Burning in eye** | Please rate the severity of any burning feelings in your eye(s) **at its worst** in the past 24 hours:  **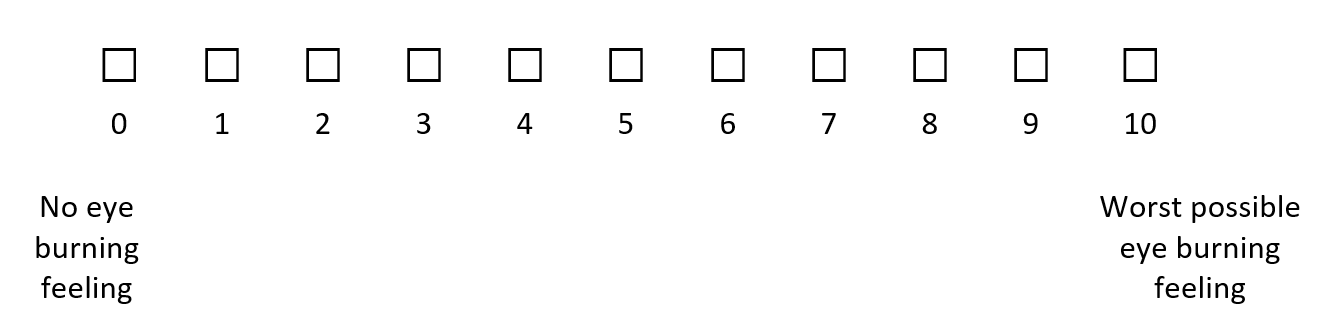** |
| **5. Eye tiredness** | Please rate the severity of your eye tiredness **at its worst** in the past 24 hours:  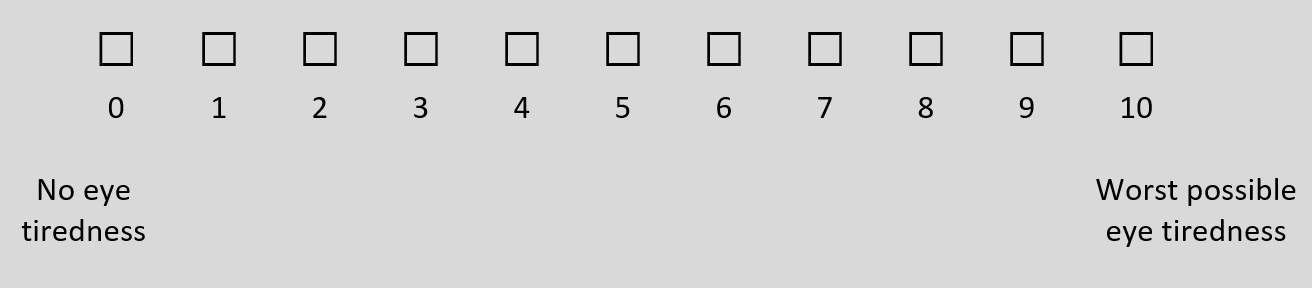 |
| **6. Feeling like there is something in your eye** | Please rate the severity of a feeling that you have something in your eye **at its worst** in the past 24 hours:  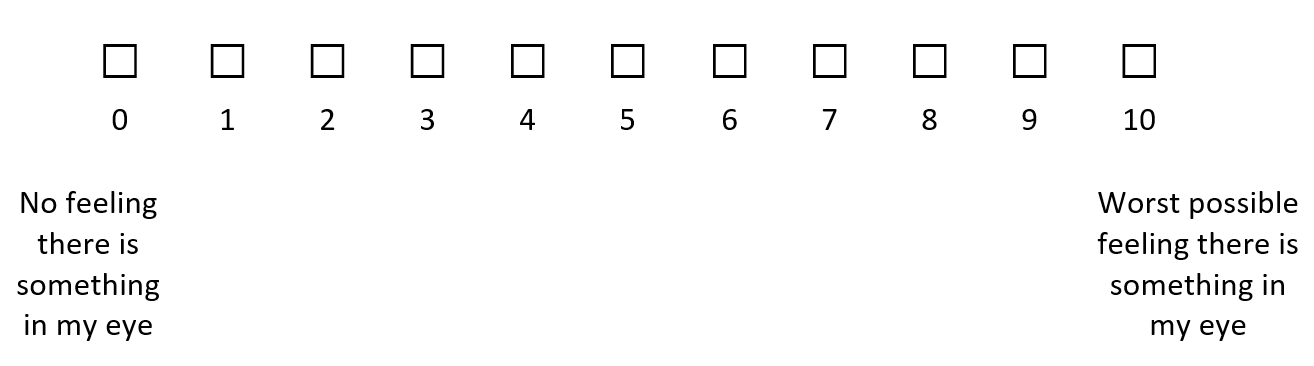 |
| **7. Eye itch** | Please rate the severity of the itch in your eye **at its worst** in the past 24 hours:  **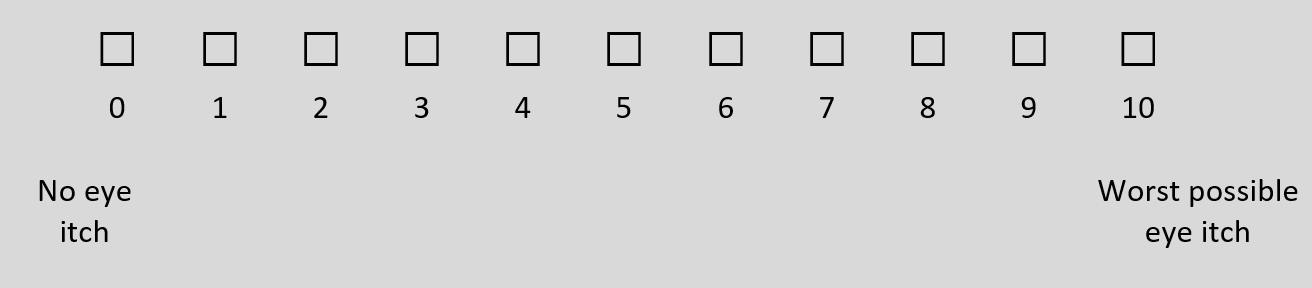** |
| **8. Eye grittiness** | Please rate the severity of a gritty feeling in your eye **at its worst** in the past 24 hours: 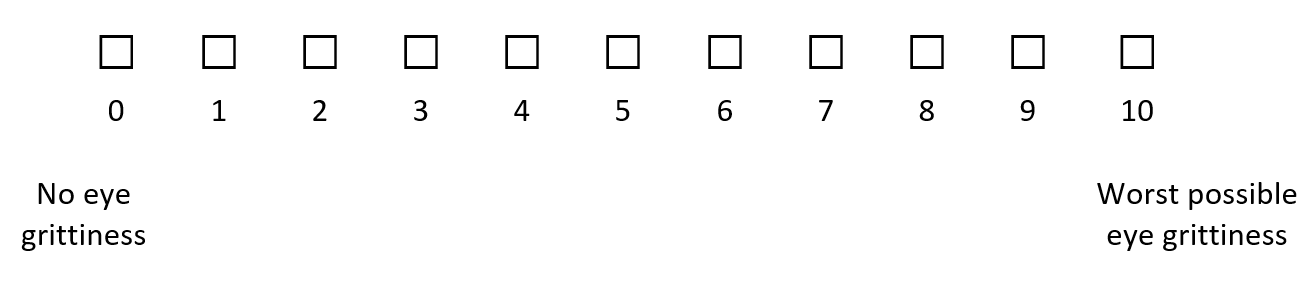 |
| **9. Mucus in or around the eye** | Please rate the severity of mucus in or around your eye **at its worst** in the past 24 hours:  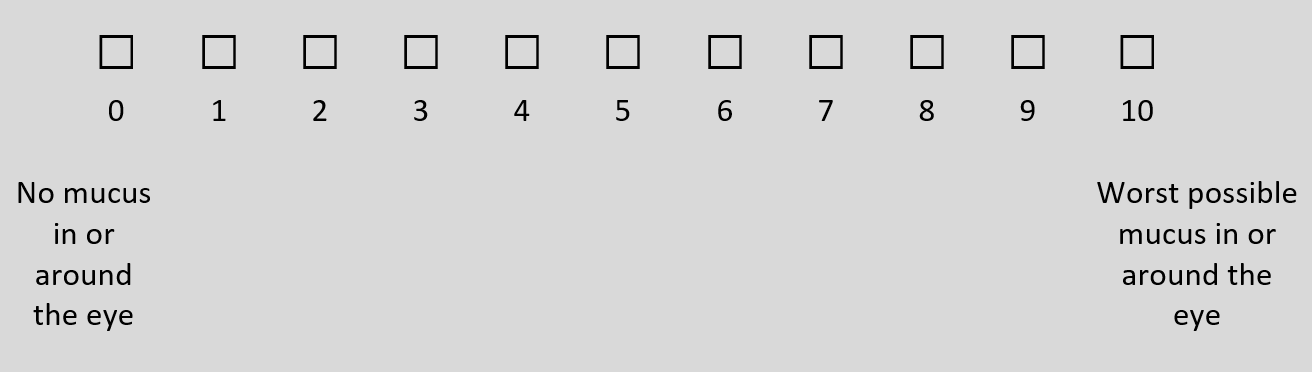 |
| **10. Eyes feeling scratched** | Please rate the severity of your eyes feeling like they have been scratched by something **at its worst** in the past 24 hours:  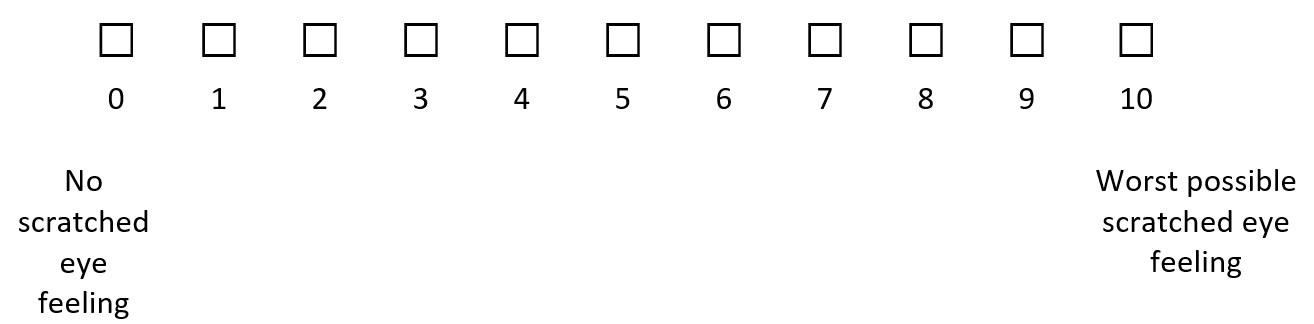 |
| **11. Eyelid redness** | Please rate the severity of your eyelid redness **at its worst** in the past 24 hours:  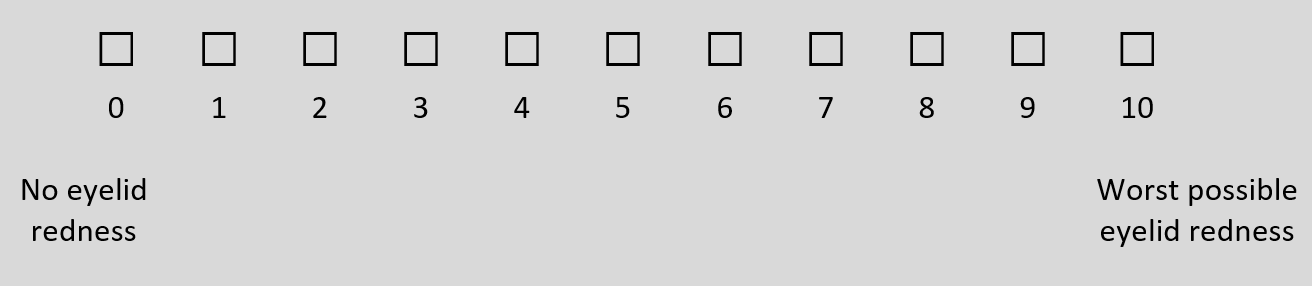 |
| **12. Eye redness** | Please rate the severity of your eye redness **at its worst** in the past 24 hours:  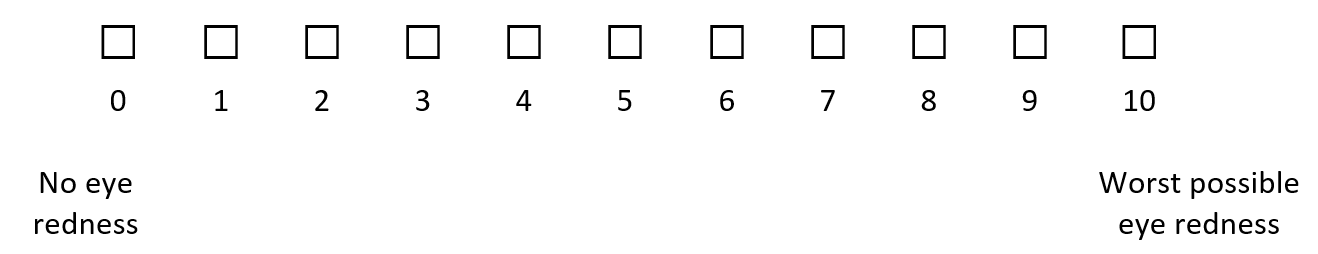 |
| **13. Watery eyes** | Please rate the severity of your watery eyes in the morning **at its worst** in the past 24 hours:  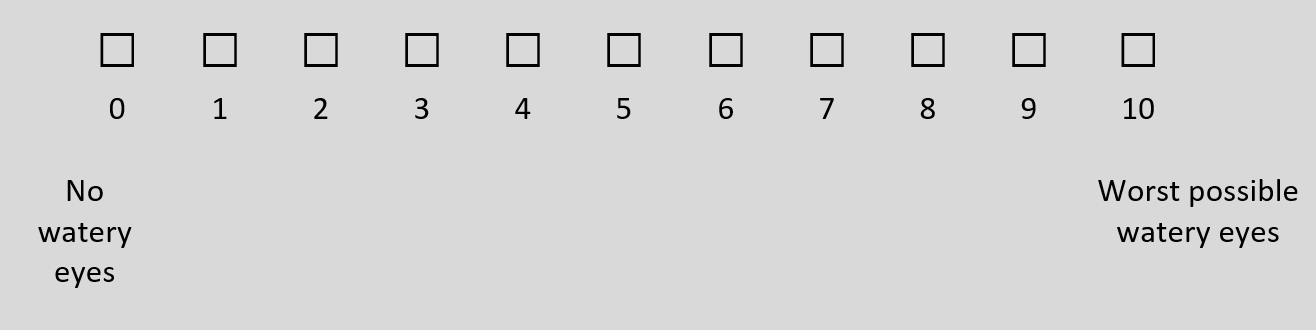 |

**Blurred Vision Module**

Please answer the following questions thinking about your blurred vision between blinks when it was **at its worst** over the past 24 hours.

| **1. Blurred vision between blinks severity** | Please rate the blurred vision you experienced between blinks **at its worst** in the past 24 hours:  **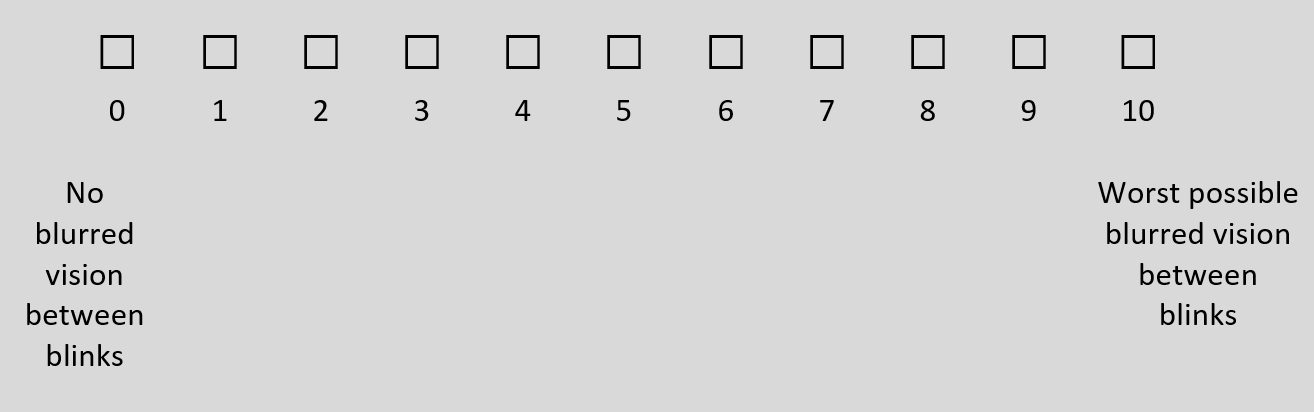** |
| --- | --- |
| **2. Blurred vision between blinks frequency** | How much of the time have you had blurred vision between blinks **in the past 24 hours**?   \| 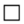 \| 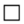 \| 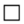 \| 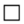 \| 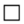 \|  \|  \| \| --- \| --- \| --- \| --- \| --- \| --- \| --- \| \| None of the time \| A little of the time \| Some of the time \| A lot of the time \| All of the time \|  \| |

**Environmental Triggers Module**

Please answer the following questions thinking about your eye dryness at the time it was **at its worst** over the past 24 hours.

| **1. Sensitivity to light** | Please rate how sensitive your eyes have been to light **at its worst** in the past 24 hours:  **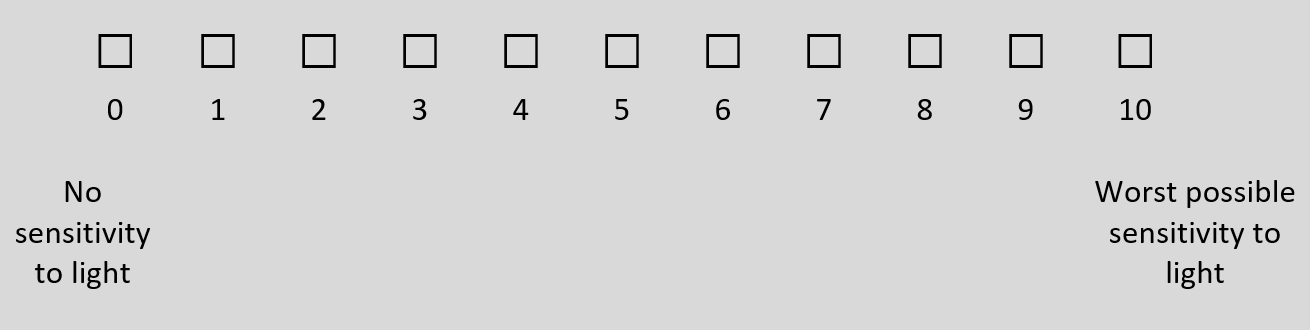** |
| --- | --- |
| **2. Sensitivity to wind** | Please rate how sensitive your eyes have been to wind **at its worst** in the past 24 hours:  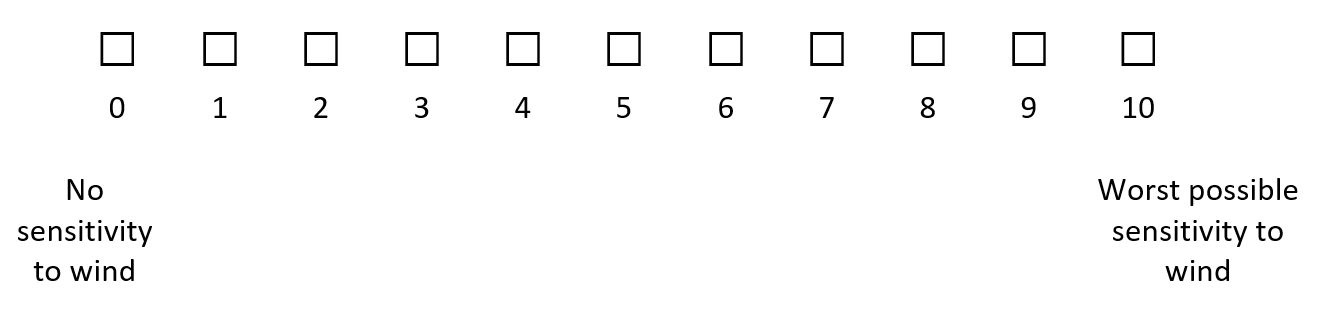 |

**Visual Tasking Module**

The following questions ask about how much of the time your **dry eye disease symptoms and related problems (e.g. blurred vision between blinks) affected or interfered with your ability to do visual activities in the past 7 days.**

Difficulties doing visual activities might include **changing how you did an activity, avoiding an activity, or needing to take a rest from an activity** because of your dry eye disease symptoms.

Please answer the questions thinking about how much of the time these activities are affected when you **do not make adjustments** (for example, think about reading on your phone **without** changing the brightness of the screen).

| Over the past 7 days how much of the time did your dry eye symptoms and related problems affect your ability to… | None of the time | A little of the time | Some of the time | A lot of the time | All of the time | I avoided or was completely unable to do this activity due to my dry eye disease symptoms | Not applicable I did not do this for reasons unrelated to my dry eye disease symptoms |
| --- | --- | --- | --- | --- | --- | --- | --- |
| 1. Read books, newspapers or magazines? |  |  |  |  |  |  |  |
| 2. Read on a screen for example a computer or tablet (without changing the brightness of the screen)? |  |  |  |  |  |  |  |
| 3. Watch a program on the TV? |  |  |  |  |  |  |  |
| 4. Carry out household chores for example cleaning or laundry? |  |  |  |  |  |  |  |
| 5. Carry out your usual leisure activities or hobbies for example crafts, painting, playing cards? |  |  |  |  |  |  |  |
| 6. Watch events at a distance for example a show or sporting event? |  |  |  |  |  |  |  |
| 7. Drive during the day? |  |  |  |  |  |  |  |
| 8. Drive at night? |  |  |  |  |  |  |  |

**Health-Related Quality of Life Module**

The following questions ask about **ways your dry eye disease symptoms may have affected you in the past 7 days.**

For each question, please choose the answer which describes how much of the time you were affected **because of your dry eye disease symptoms in the past 7 days**.

| **Over the past 7 days because of your dry eye disease symptoms…** | | **None of the time** | **A little of the time** | **Some of the time** | **A lot of the time** | **All of the time** |
| --- | --- | --- | --- | --- | --- | --- |
| **1** | How much of the time did you feel low or depressed? |  |  |  |  |  |
| **2** | How much of the time did you feel anxious? |  |  |  |  |  |
| **3** | How much of the time did you feel frustrated? |  |  |  |  |  |
| **4** | How much of the time did you feel worried? |  |  |  |  |  |
| **5** | How many nights did your dry eye disease symptoms affect your sleep? | - 0 nights - 1-2 nights - 3-4 nights - 5-6 nights - Every night | | | | |

# PGI items v2_0 debriefed in round 1 patient interviews

**Global Impression of Severity**

**1.** Please choose the response below that best describes the overall severity of your **eye dryness** over the **past 7 days.**

□ Severe

□ Moderate

□ Mild

□ None

**2.** Please choose the response below that best describes the overall severity of your **dry eye disease symptoms** over the **past 7 days.**

□ Severe

□ Moderate

□ Mild

□ None

**3.** Please choose the response below that best describes the overall severity of your **limitations in carrying out visual activities (e.g. reading, watching the TV, driving)** **due to dry eye disease symptoms and related problems** over the **past 7 days.**

□ Severe

□ Moderate

□ Mild

□ None

**Global Impression of Change**

**1.** Please choose the response below that best describes the overall change in your **eye dryness** compared to when you started taking the study treatment.

□ Much better

□ A little better

□ No change

□ A little worse

□ Much worse

**2.** Please choose the response below that best describes the overall change in your **dry eye disease symptoms** compared to when you started taking the study treatment.

□ Much better

□ A little better

□ No change

□ A little worse

□ Much worse

**3.** Please choose the response below that best describes the overall change in your **limitations in carrying out visual activities (e.g. reading, watching the TV, driving) due to your dry eye disease symptoms and related problems** compared to when you started taking the study treatment (select one response).

□ Much better

□ A little better

□ No change

□ A little worse

□ Much worse

# DED-Q v4_0 debriefed in round 2 HCP and patient interviews

**Eye Dryness Severity Module**

| **1.** | 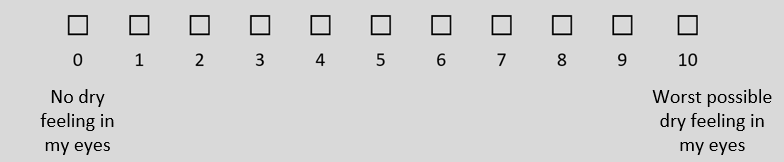Please rate the severity of your eyes feeling dry **right now**: |
| --- | --- |

**Eye Dryness Frequency Module**

| **1.** | How much of the time have your eyes felt dry **in the past 24 hours**?   \| 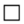 \| 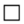 \| 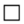 \| 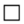 \| 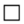 \| \| --- \| --- \| --- \| --- \| --- \| \| None of the time \| A little of the time \| Some of the time \| A lot of the time \| All of the time \| |
| --- | --- | --- | --- | --- | --- | --- | --- | --- | --- | --- | --- |

**Dry Eye Disease Severity Module**

| **1.** | 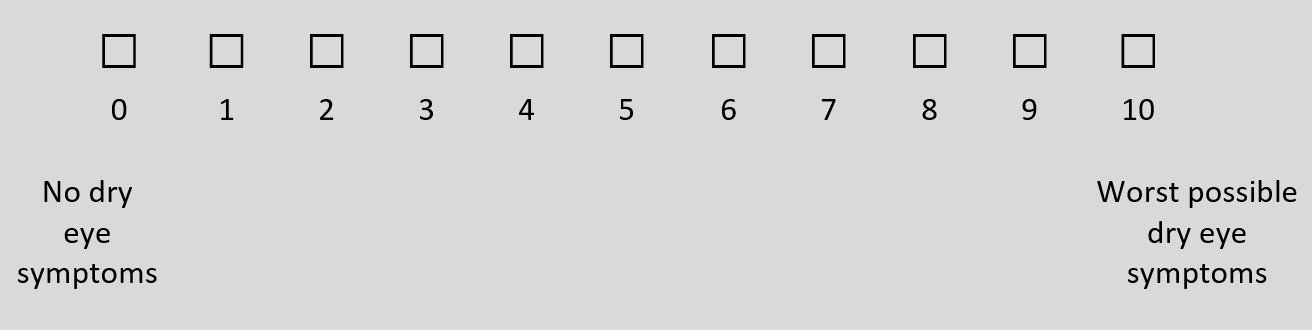Please rate the severity of your dry eye disease symptoms **right now**: |
| --- | --- |

**Dry Eye Disease Frequency Module**

| **1.** | How much of the time have you had dry eye disease symptoms **in the past 24 hours**?   \| 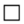 \| 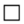 \| 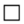 \| 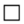 \| 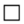 \| \| --- \| --- \| --- \| --- \| --- \| \| None of the time \| A little of the time \| Some of the time \| A lot of the time \| All of the time \| |
| --- | --- | --- | --- | --- | --- | --- | --- | --- | --- | --- | --- |

**Symptom Module**

Please answer the following questions thinking about each symptom when it was **at its worst** over the past 24 hours.

| **1. Eyes feeling dry** | 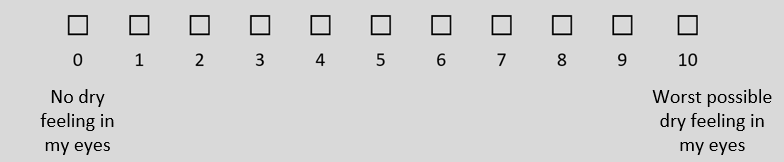Please rate the severity of your eyes feeling dry **at its worst** in the past 24 hours: |
| --- | --- |
| **2. Eye pain** | 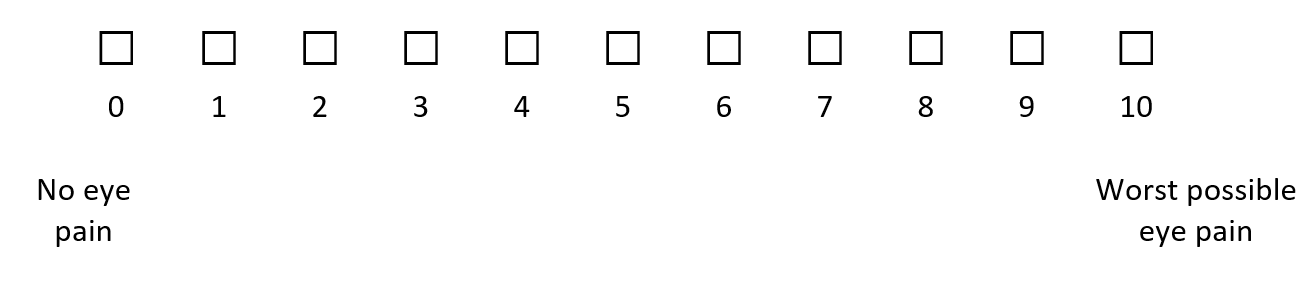Please rate the severity of your eye pain **at its worst** in the past 24 hours: |
| **3. Eye irritation** | **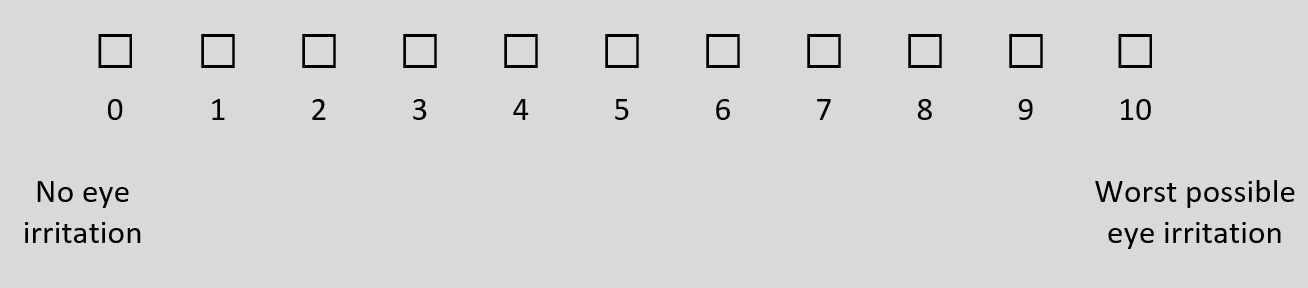**Please rate the severity of your eye irritation **at its worst** in the past 24 hours: |
| **4. Burning** | **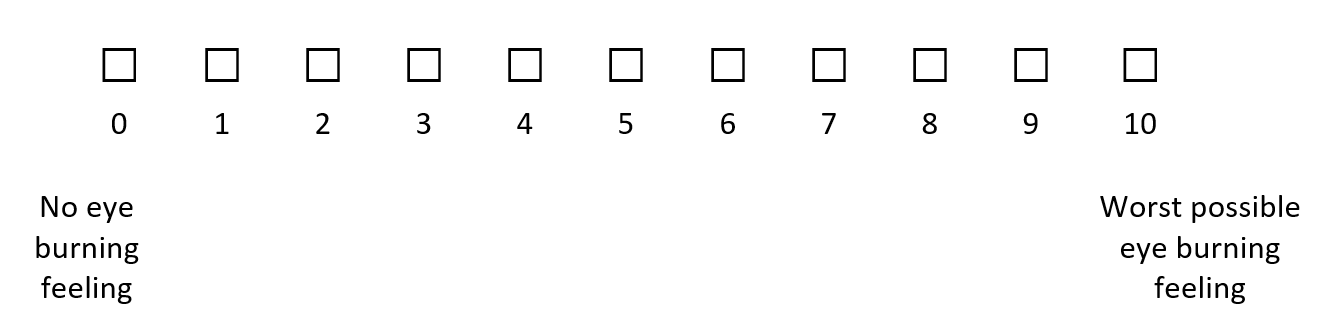**Please rate the severity of any burning feelings in your eye(s) **at its worst** in the past 24 hours: |
| **5.Eye tiredness** | 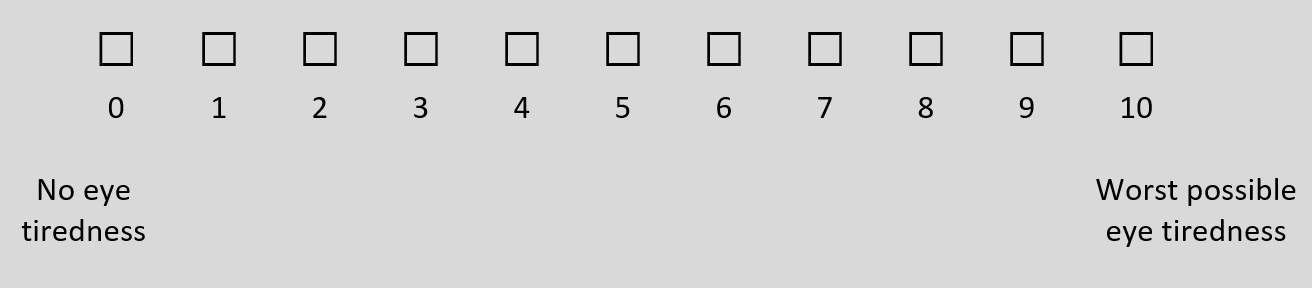Please rate the severity of your eye tiredness **at its worst** in the past 24 hours: |
| **6. Feeling like there is something in your eye** | 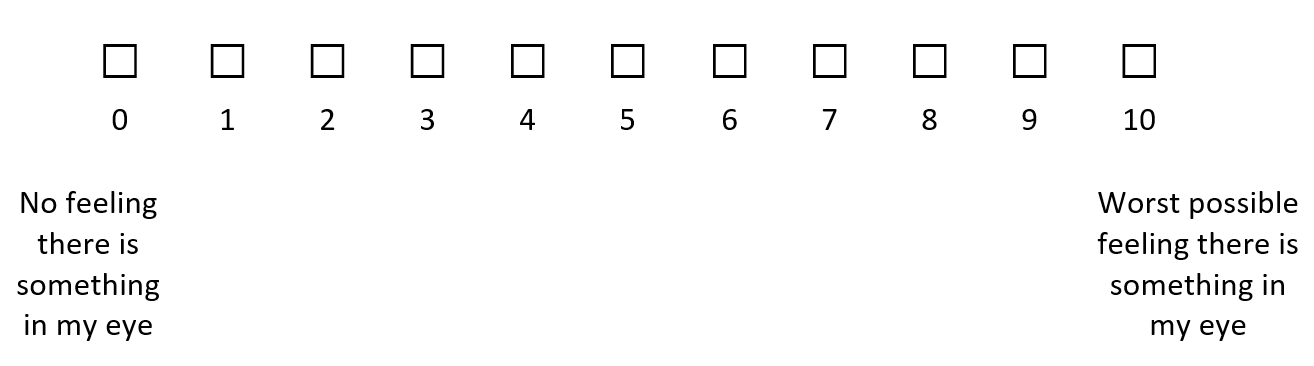Please rate the severity of a feeling that you have something in your eye **at its worst** in the past 24 hours: |
| **7. Eye itch** | **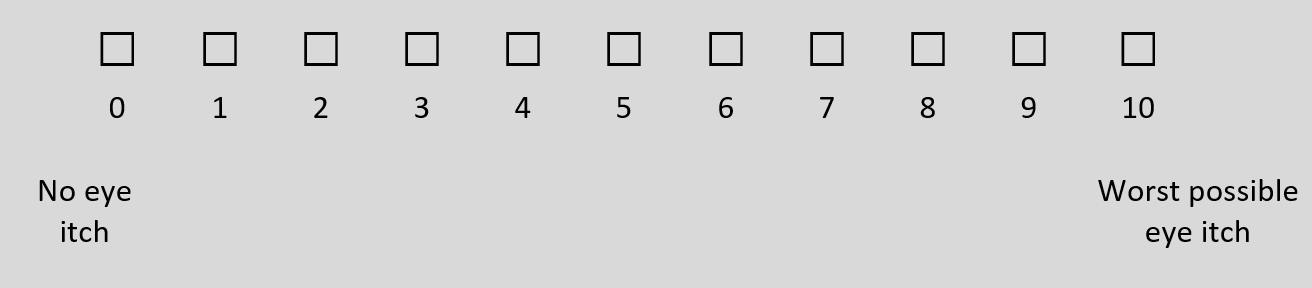**Please rate the severity of the itch in your eye **at its worst** in the past 24 hours: |
| **8. Eye grittiness** | 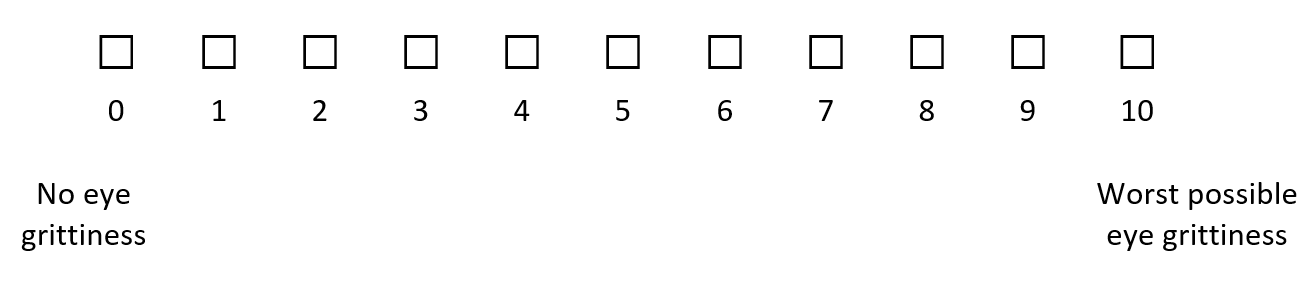Please rate the severity of a gritty feeling in your eye **at its worst** in the past 24 hours: |
| **9. Mucus in or around your eye** | 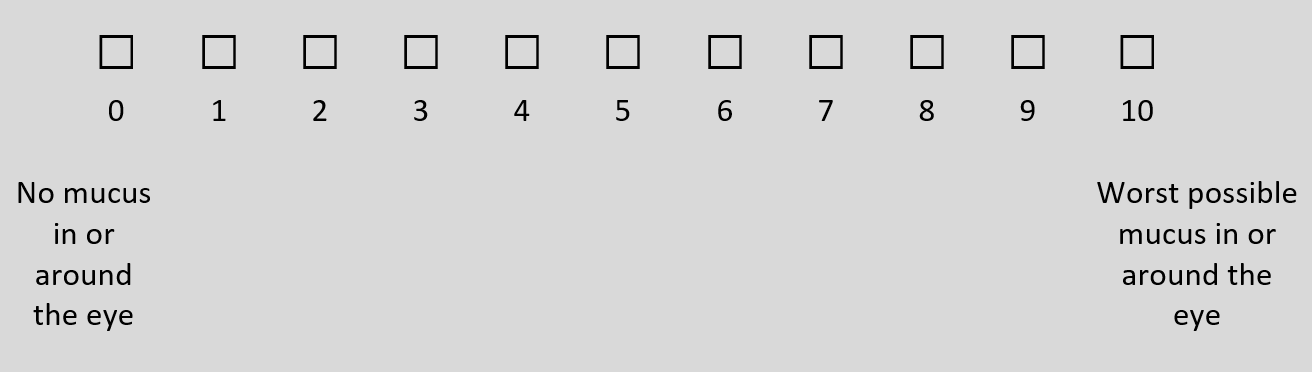Please rate the severity of mucus in or around your eye **at its worst** in the past 24 hours: |
| **10. Eyes feeling scratched** | 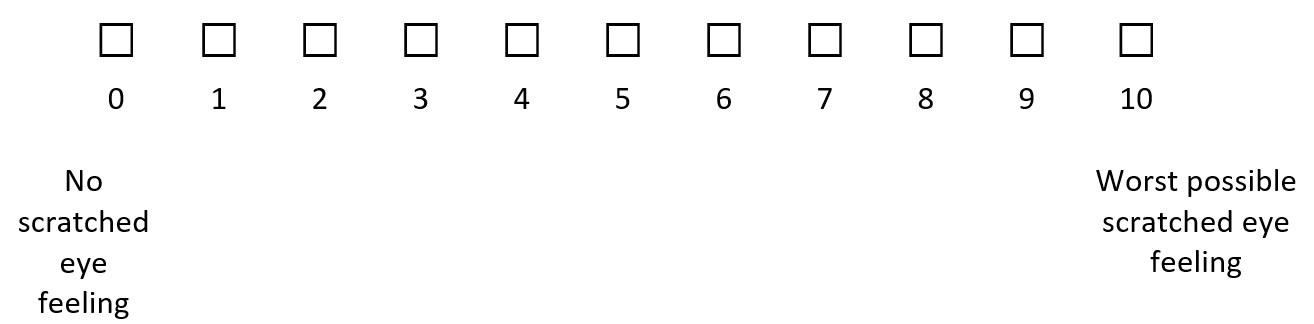Please rate the severity of your eyes feeling like they have been scratched by something **at its worst** in the past 24 hours: |
| **11. Eyelid redness** | 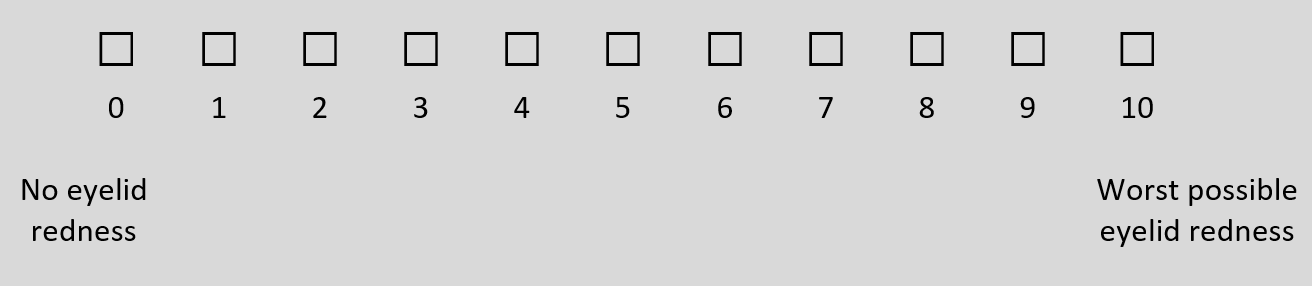Please rate the severity of your eyelid redness **at its worst** in the past 24 hours: |
| **12. Eyeball redness** | 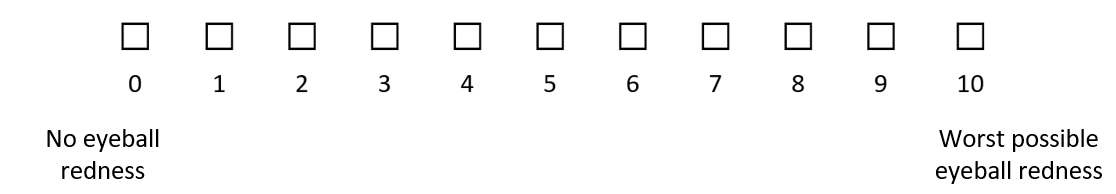Please rate the severity of your eyeball redness **at its worst** in the past 24 hours: |
| **13. Watery eyes** | 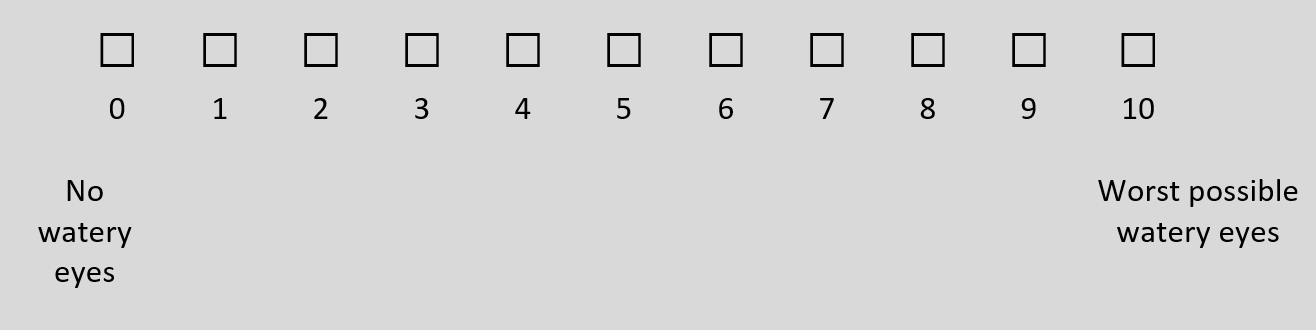Please rate the severity of your watery eyes in the morning **at its worst** in the past 24 hours: |

**Blurred Vision Module**

Please answer the following questions thinking about your blurred vision between blinks when it was **at its worst** over the past 24 hours.

| **1.** | **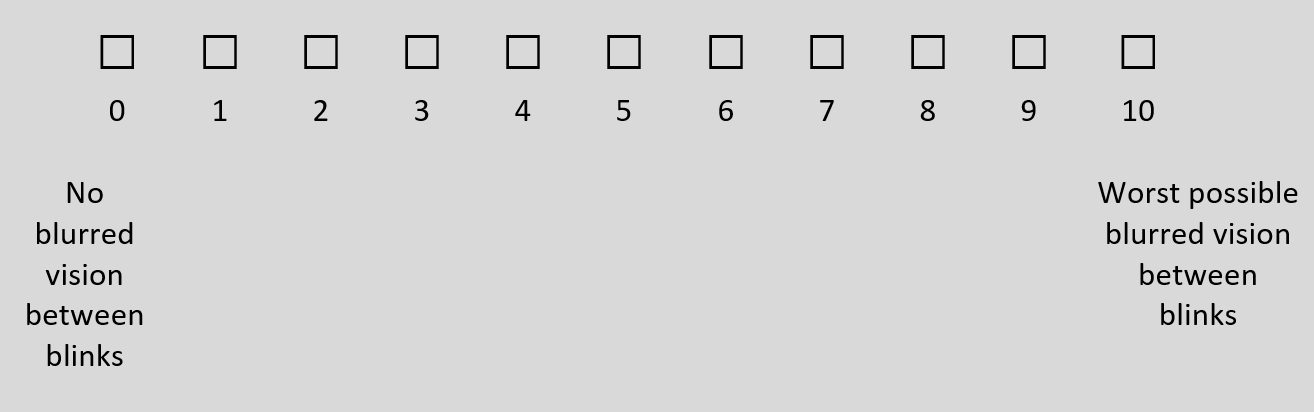**Please rate the blurred vision you experienced between blinks **at its worst** in the past 24 hours: |
| --- | --- |
| **2.** | How much of the time have you had blurred vision between blinks **in the past 24 hours**?     \| 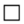 \| 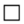 \| 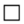 \| 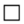 \| 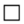 \| \| --- \| --- \| --- \| --- \| --- \| \| None of the time \| A little of the time \| Some of the time \| A lot of the time \| All of the time \| |

**Visual Tasking Module**

The following questions ask about how much of the time your **dry eye disease symptoms and related problems (e.g., blurred vision between blinks) affected or interfered with your ability to do visual activities in the past 7 days.** Please do not think about any other vision problems you have (such as vision problems that are corrected by wearing glasses, reading glasses, or contact lenses) when selecting an answer.

Difficulties doing visual activities might include **changing how you did an activity, avoiding an activity, or needing to take a rest from an activity** because of your dry eye disease symptoms.

Please answer the questions thinking about how much of the time these activities are affected by your dry eye disease when you **do not make adjustments** (for example, if your eyes are sensitive to light, think about reading on your computer or tablet **without** changing the brightness of the screen).

| Over the past 7 days how much of the time did your dry eye symptoms and related problems affect your ability to… | None of the time | A little of the time | Some of the time | A lot of the time | All of the time | I avoided or was completely unable to do this activity due to my dry eye disease symptoms | Not applicable I did not do this for reasons unrelated to my dry eye disease symptoms |
| --- | --- | --- | --- | --- | --- | --- | --- |
| 1. Read books, newspapers or magazines? |  |  |  |  |  |  |  |
| 2. Read on a screen for example a computer or tablet (without changing the brightness of the screen)? |  |  |  |  |  |  |  |
| 3. Watch a program on the TV? |  |  |  |  |  |  |  |
| 4. Carry out household chores for example cleaning or laundry? |  |  |  |  |  |  |  |
| 5. Carry out your usual leisure activities or hobbies for example crafts, painting, playing cards? |  |  |  |  |  |  |  |
| 6. Watch events at a distance for example a show or sporting event? |  |  |  |  |  |  |  |
| 7. Drive during the day? |  |  |  |  |  |  |  |
| 8. Drive at night? |  |  |  |  |  |  |  |

**Health-Related Quality of Life Module**

The following questions ask about **ways your dry eye disease symptoms may have affected you in the past 7 days.**

For each question, please choose the answer which describes how much of the time you were affected **because of your dry eye disease symptoms in the past 7 days**.

| **Over the past 7 days because of your dry eye disease symptoms…** | **None of the time** | **A little of the time** | **Some of the time** | **A lot of the time** | **All of the time** |
| --- | --- | --- | --- | --- | --- |
| 1. How much of the time did you feel low or depressed? |  |  |  |  |  |
| 2. How much of the time did you feel anxious? |  |  |  |  |  |
| 3. How much of the time did you feel frustrated? |  |  |  |  |  |
| 4. How much of the time did you feel worried? |  |  |  |  |  |
| 5. How many nights did your dry eye disease symptoms affect your sleep? | - 0 nights - 1-2 nights - 3-4 nights - 5-6 nights - Every night | | | | |

# PGI-S and PGI-C v3_0 debriefed in round 2 patient interviews

**Global Impression of Severity**

**1.** Please choose the response below that best describes the overall severity of your **eyes feeling dry** over the **past 7 days.**

□ Severe

□ Moderate

□ Mild

□ None

**2.** Please choose the response below that best describes the overall severity of your **dry eye disease symptoms** over the **past 7 days.**

□ Severe

□ Moderate

□ Mild

□ None

**3.** Please choose the response below that best describes the overall severity of your **limitations in carrying out visual activities (e.g. reading, watching the TV, driving)** **due to dry eye disease symptoms and related problems** over the **past 7 days.**

□ Severe

□ Moderate

□ Mild

□ None

**Global Impression of Change**

**1.** Please choose the response below that best describes the overall change in your **eyes feeling dry** compared to when you started taking the study treatment.

□ Much better

□ A little better

□ No change

□ A little worse

□ Much worse

**2**. Please choose the response below that best describes the overall change in your **dry eye disease symptoms** compared to when you started taking the study treatment.

□ Much better

□ A little better

□ No change

□ A little worse

□ Much worse

**3.** Please choose the response below that best describes the overall change in your **limitations in carrying out visual activities (e.g. reading, watching the TV, driving) due to your dry eye disease symptoms and related problems** compared to when you started taking the study treatment (select one response).

□ Much better

□ A little better

□ No change

□ A little worse
