# Supplementary material for: Development and content validity assessment of the Dry Eye Disease Questionnaire in patients with dry eye disease, meibomian gland dysfunction, and Sjögren’s syndrome dry eye disease
Source: J Patient Rep Outcomes. 2023 Jul 5;7:64. doi: 10.1186/s41687-023-00608-5 (PMC10323053; doi:10.1186/s41687-023-00608-5)
Supplement: Supplementary file 4 — Additional file 4. CD interviews: Participant understanding and relevance findings for the DED-Q modules. [file 41687_2023_608_MOESM4_ESM.docx]

# Patient understanding of the DED-Q items

EDM = Eye Dryness Module; DEDSM = Dry Eye Disease Symptom Module; SM = Symptom Module

Figure 1. Understanding of the DED-Q Eye Dryness, Dry Eye Disease Symptom, and Symptom Modules in DED, MGD, and SS-DED patients

Figure 2. Understanding of the Blurred Vision Module in DED, MGD, and SS-DED patients

Figure 3. Understanding of the Environmental Triggers Module in DED, MGD, and SS-DED patients

**Figure 4. Understanding of the Visual Tasking Module in DED, MGD, and SS-DED patients**

Figure 5. Understanding of the HRQoL Module in DED, MGD, and SS-DED patients

### 2. Concept relevance in DED, MGD, and SS-DED patient interviews of the DED-Q

EDM = Eye Dryness Module; DEDSM = Dry Eye Disease Symptom Module

**Figure 6. Relevance of the DED-Q Eye Dryness and Dry Eye Disease Symptom Modules in DED, MGD, and SS-DED patients**

Figure(s) 7. Relevance of the Symptom Module in DED, MGD, and SS-DED patients

Figure 8. Relevance of the Blurred Vision Module in DED, MGD, and SS-DED patients

Figure 9. Relevance of the Environmental Triggers Module in DED, MGD, and SS-DED patients (Round 1 only)

**Figure 10. Relevance of the DED-Q Visual Tasking Module in DED, MGD, and SS-DED patients**

Figure 11. Relevance of the HRQoL Module in DED, MGD, and SS-DED patients
